# Experimental Determination of Air/Water Partition Coefficients for 21 Per- and Polyfluoroalkyl Substances Reveals Variable Performance of Property Prediction Models

**DOI:** 10.1021/acs.est.3c02545

**Published:** 2023-05-26

**Authors:** Satoshi Endo, Jort Hammer, Sadao Matsuzawa

**Affiliations:** Health and Environmental Risk Division, National Institute for Environmental Studies (NIES), Onogawa 16-2, 305-8506 Tsukuba, Ibaraki, Japan

**Keywords:** Henry’s law constant, hexadecane/water partition coefficient, thermodynamic cycle, shared-headspace, variable phase ratio headspace method, COSMO*therm*, fluorotelomer substance, perfluoroalkane sulfonamide

## Abstract

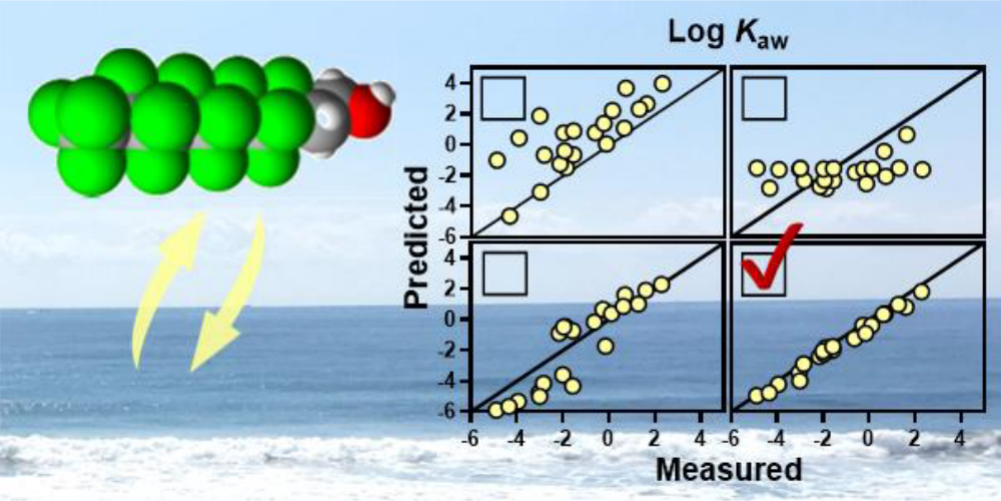

Per- and polyfluoroalkyl substances (PFAS) are a group of chemicals of high environmental concern. However, reliable data for the air/water partition coefficients (*K*_aw_), which are required for fate, exposure, and risk analysis, are available for only a few PFAS. In this study, *K*_aw_ values at 25 °C were determined for 21 neutral PFAS by using the hexadecane/air/water thermodynamic cycle. Hexadecane/water partition coefficients (*K*_Hxd/w_) were measured with batch partition, shared-headspace, and/or modified variable phase ratio headspace methods and were divided by hexadecane/air partition coefficients (*K*_Hxd/air_) to obtain *K*_aw_ values over 7 orders of magnitude (10^–4.9^ to 10^2.3^). Comparison to predicted *K*_aw_ values by four models showed that the quantum chemically based COSMO*therm* model stood out for accuracy with a root-mean-squared error (RMSE) of 0.42 log units, as compared to HenryWin, OPERA, and the linear solvation energy relationship with predicted descriptors (RMSE, 1.28–2.23). The results indicate the advantage of a theoretical model over empirical models for a data-poor class like PFAS and the importance of experimentally filling data gaps in the chemical domain of environmental interest. *K*_aw_ values for 222 neutral (or neutral species of) PFAS were predicted using COSMO*therm* as current best estimates for practical and regulatory use.

## Abstract 2

New data for air/water partition coefficients for 21 PFAS demonstrated accurate prediction by quantum chemically based COSMO*therm* and lower performance of empirical fit models.

## 1 Introduction

Per- and polyfluoroalkyl substances (PFAS) are a group of chemicals with high environmental concern because they are either resistant to degradation or transform into substances that are persistent in the environment and ecosystems. Some PFAS [e.g., perfluorooctane sulfonate (PFOS), perfluorooctanoic acid (PFOA)] have been proven to have significant health influences on some populations at the current levels of contamination.^1^ Since accumulation of PFAS in humans and animals was reported in 2001,^2−5^ active research has been conducted on this class of chemicals worldwide. According to Bakhshoodeh and Santos,^6^ the annual number of scientific publications on PFAS exceeded 100 in 2007 and reached ∼900 in 2020. Despite the proliferation of literature on PFAS, experimental data for basic physicochemical properties, including air/water partition coefficients (*K*_aw_), are scarce. *K*_aw_ largely dictates the contaminant transfer between air and water and has a tremendous influence on the fate of the chemical in the environment. In addition, *K*_aw_ has implications for analytical methods (e.g., sample collection, storage, extraction), remediation techniques (e.g., air stripping), and ecotoxicological test settings suitable for given chemicals.

There have been only a few scientific articles that reported direct measurements of *K*_aw_ for PFAS with a perfluorocarbon chain length of 4 or more. Three studies measured *K*_aw_ for 3 or 4 fluorotelomer alcohols (FTOHs).^7−9^ Some of the reported values, however, differ between the studies by up to a factor of 130 for a single compound. Two other studies took on the challenge of measuring *K*_aw_ of the neutral species of the strong acid PFOA and provided values that are an order of magnitude different.^10,11^ To fill the huge data gap, various researchers derived *K*_aw_ from the saturated vapor pressure relative to the aqueous solubility (VP/S).^8,9,12,13^ Even including such indirectly measured *K*_aw_ data, the authors of a recent review^13^ listed experimental data for only seven PFAS. A reason for the data limitation could be that PFAS of major concern are strong acids, which ionize and become strongly soluble in water, making *K*_aw_ of the neutral species less important for environmental fate analysis. Nevertheless, there exist many neutral or largely neutral PFAS, some of which are known precursors of the PFAS of high concern (i.e., PFOS, PFOA).^14−16^ Another reason may be measurement difficulty associated with the interfacial active nature of many PFAS. Direct measurement of *K*_aw_ often requires quantification of the gas phase PFAS concentrations^11,17^ or relies on the mass balance in a closed air/water system,^7,8^ neither of which could conveniently be achieved if the adsorption to a third phase such as a glass wall or air/water interface is significant. As *K*_aw_ data are largely missing, environmental transport models inevitably use model-based predictions.^18,19^ However, no experimental data means no model calibration and validation; indeed, Arp et al.^20^ reported prediction errors of several orders of magnitude in *K*_aw_ by some models.

The objectives of this study are to obtain new experimental data for *K*_aw_ at 25 °C for a series of neutral PFAS and to evaluate *K*_aw_ prediction models. New data for PFAS with various molecular structures can also improve our understanding on the structure–property relationship. To circumvent the difficulty associated with direct measurement of *K*_aw_, we determined *K*_aw_ indirectly by measuring the hexadecane/water partition coefficient (*K*_Hxd/w_) and applying the hexadecane/air/water thermodynamic cycle:
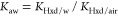
1where *K*_Hxd/air_ is the hexadecane/air partition coefficient. *K*_Hxd/w_ can be measured more reliably than *K*_aw_ because gas phase quantification is not needed and hexadecane has a larger solubilization capacity than water, which minimizes the problem of third-phase sorption. Experimental *K*_Hxd/air_ values for 61 PFAS have already been reported in our previous study.^21^ A similar approach could be taken with the octanol/air/water cycle. However, octanol/air partition coefficients are unavailable for most PFAS. Additionally, there is some mutual solubility between octanol and water, and water-saturated octanol has been shown to have slightly different properties than pure octanol.^22^ In contrast, hexadecane has very little mutual solubility with water and is an ideal solvent to be used for the thermodynamic cycle approach.^23^ The new *K*_aw_ data for 21 PFAS were compared to predictions by four models that are often used in environmental risk assessments. *K*_aw_ prediction models have been evaluated against experimental data in the literature but only for four FTOHs^20,24^ and four additional PFAS^13^ so far because of the data limitation.

## 2 Materials and Methods

### 2.1 Chemicals

*K*_Hxd/w_ values were measured for 21 PFAS; their names, abbreviations, and CAS registry numbers are listed in Table 1. These compounds were selected according to their environmental relevance, structural variation, and measurement possibility. The providers and purity of the reagents are provided in Table S1, Supporting Information (SI). Hexadecane (anhydrous, ≥99%) was purchased from Sigma-Aldrich (Tokyo, Japan). Methanol, acetone, and *n*-hexane were of analytical grade and from Fujifilm Wako Chemicals (Osaka, Japan). Reverse osmosis-treated tap water was further purified with an Ultrapure Water System (RFU665DA, Advantec, Tokyo, Japan).

**Table 1:** List of PFAS Used in This Study^a^

name	abbreviation	CAS-RN
1H,1H-perfluorobutan-1-ol	3:1 FTOH	375-01-9
3-(perfluoropropyl)propan-1-ol	3:3 FTOH	679-02-7
1H,1H,2H,2H-perfluorohexan-1-ol	4:2 FTOH	2043-47-2
4-(perfluorobutyl)butan-1-ol	4:4 FTOH	3792-02-7
1H,1H,2H,2H-perfluorooctan-1-ol	6:2 FTOH	647-42-7
1H,1H-perfluorooctan-1-ol	7:1 FTOH	307-30-2
1H,1H,2H,2H-perfluorodecan-1-ol	8:2 FTOH	678-39-7
1H,1H,1H,2H-perfluoroheptan-2-ol	5:2s FTOH	914637-05-1
1H,1H,2H,2H-perfluorohexyl iodide	4:2 FTI	2043-55-2
1H,1H-perfluoroheptyl iodide	6:1 FTI	212563-43-4
1H,1H,7H-perfluoroheptyl iodide	6:1 FTI-7H	376-32-9
1H,1H,2H,2H-perfluorooctyl acrylate	6:2 FTAC	17527-29-6
1H,1H,2H,2H-perfluorohexyl methacrylate	4:2 FTMAC	1799-84-4
perfluorobutane sulfonamide	PFBSA	30334-69-1
perfluorohexane sulfonamide	PFHxSA	41997-13-1
perfluorooctane sulfonamide	PFOSA	754-91-6
*N*-methyl perfluorobutane sulfonamide	MeFBSA	68298-12-4
*N*-methyl perfluorohexane sulfonamide	MeFHxSA	68259-15-4
*N*-ethyl perfluorohexane sulfonamide	EtFHxSA	87988-56-5
*N*-methyl perfluorobutane sulfonamidoethanol	MeFBSE	34454-97-2
*N*-ethyl perfluorohexane sulfonamidoethanol	EtFHxSE	34455-03-3

a See Table S2 for the molecular structure represented by SMILES strings.

### 2.2 Experimental Determination of *K*_Hxd/w_

*K*_Hxd/w_ values were determined by batch partition, shared-headspace, and/or modified variable phase ratio headspace (VPR-HS) methods. The experimental procedures for these methods are described in full in SI-1; here, brief explanations are provided.

#### 2.2.1 Batch Partition Method

Hexadecane solution of PFAS (0.1–2000 mg/L) and water were put in 10-mL glass vials and gently shaken for 24 h (60 rpm, 25 °C). An aliquot of the water phase was analyzed by liquid chromatography/mass spectrometry (LC/MS; for all eight sulfonamides) or liquid–liquid-extracted with *n*-hexane and analyzed by gas chromatography (GC)/MS (for all others). The conditions for LC/MS and GC/MS analyses are described in SI-2. *K*_Hxd/w_ was calculated using the measured water phase concentration under the mass conservation assumption. The 95% confidence interval (CI) of the mean (*x*) was calculated with the formula, *x* ± 2.78 SD/√5, where SD is the standard deviation, based on the *t*-distribution and *n* = 5. The hexadecane phase was also analyzed with LC/MS or GC/MS, which confirmed 93–112% of the expected concentrations present in hexadecane. The PFAS concentration prepared in hexadecane and volumes of water and hexadecane, and recovery from the hexadecane phase for each compound are given in Table S3. The batch partition method was performed for 18 PFAS. It was not performed for 3:1 FTOH because its extraction from water and GC-separation from the solvent peak was difficult by the current method. Moreover, 7:1 and 8:2 FTOHs were not measured, as the shared-headspace method was expected to provide more accurate values of *K*_Hxd/w_ for these relatively hydrophobic chemicals (see the Results section).

For unsubstituted perfluoroalkane sulfonamides (PFASAs) and their *N*-alkyl derivatives, namely, PFBSA, PFHxSA, PFOSA, MeFBSA, MeFHxSA, and EtFHxSA, 1 mM HCl aqueous solution instead of pure water was used. Preliminary experiments with PFBSA and PFHxSA with pure water resulted in log *K*_Hxd/w_ values that were 0.5–0.9 log units lower than those determined with 1 mM HCl solution later. While measured p*K*_a_ values of these compounds are unavailable, an experimental p*K*_a_ value of 6.33 was reported for trifluoromethane sulfonamide (CF_3_-SO_2_NH_2_).^25^ COSMO*therm* predicted p*K*_a_ values of 6.4 and 6.3 for PFBSA and PFHxSA, respectively, and 7.3 and 7.2 for MeFBSA and MeFHxSA, respectively. These results suggest that PFASAs and their *N*-alkyl compounds can be deprotonated in unbuffered water but remain largely neutral in 1 mM HCl.

#### 2.2.2 Shared-Headspace Method

The method concept is based on the existing publications that used a shared-headspace system for salting-out experiments^26^ and ecotoxicity tests.^27,28^ Hexadecane solution of PFAS was placed in a 350 μL insert hosted by a 1.5 mL GC vial. This small vial was put in a 10 mL headspace vial that contained 1 mL of water. The 10 mL vial was closed with a PTFE-lined septum and gently shaken at 25 °C for 24 h. During this time, the compound dissolved in hexadecane was partially transferred to water via the headspace until a three-phase partition equilibrium was reached between hexadecane, air, and water. Water was then sampled and immediately extracted with *n*-hexane for GC/MS analysis. The hexadecane phase was also analyzed, which confirmed 85–100% mass conservation (Table S3). *K*_Hxd/w_ was obtained from the measured concentration in water, as in the batch partition method. The shared-headspace approach does not require a direct contact between water and hexadecane, avoiding the possible formation of emulsions or microdroplets in water. The shared-headspace method is useful for compounds with sufficient volatility (relatively high *K*_aw_, low *K*_Hxd/air_) and hydrophobicity (relatively high *K*_Hxd/w_), as these properties ensure an efficient transfer from hexadecane to water via air and negligible concentration depletion in hexadecane. Therefore, nine PFAS that meet this requirement were measured by this method, while all eight sulfonamides and four short-chain FTOHs were not.

#### 2.2.3 Modified VPR-HS Method

*K*_Hxd/w_ was also measured with a modified version of the VPR-HS method; 20 mL headspace vials were filled with water and hexadecane with different volume ratios. The total liquid volume was always 20 mL. All vials received the same amount of the target compound. After equilibration at 25 °C, the headspace was injected into the GC/MS. Based on the mass balance, the peak area (PA) should have the following relationship with *K*_Hxd/w_:
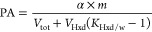
2where α is the GC/MS response factor, *m* is the mass of the compound added to the vial, *V*_tot_ is the total liquid volume, and *V*_Hxd_ is the volume of hexadecane solution. Here, response linearity as well as no significant mass of the compound in the headspace and any other interface (e.g., vial wall, hexadecane/water interface, septum) was assumed. As α, *m*, and *V*_tot_ (=20 mL) are constant for a given compound, *K*_Hxd/w_ can be fitted on the data of PA vs *V*_Hxd_. Theoretically, *K*_Hxd/w_ could be determined with a minimum of two *V*_Hxd_ conditions (e.g., 0 and 20 mL), whereas at least four *V*_Hxd_ values were prepared in this work. Five FTOHs were measured with this method, which were measurable by headspace GC/MS and expected to have *K*_Hxd/w_ that is low enough for this method.

### 2.3 Direct Measurement of *K*_aw_ Using the VPR-HS Method

*K*_aw_ values for five PFAS were directly measured using a standard VPR-HS method, as in the literature.^7,8^ The experimental procedure is similar to what has been described previously for *K*_Hxd/air_(21) and is summarized in SI-3. This measurement was conducted for a comparison purpose because the VPR-HS measurement for *K*_aw_ could result in substantial errors if the mass balance assumption does not hold true, as was experimentally demonstrated before.^8^

### 2.4 Prediction Models for *K*_aw_

In this work, four prediction models for *K*_aw_ were considered. All need only the molecular structure as an input parameter.

#### 2.4.1 HenryWin

HenryWin (v3.21, April 2015) is a module of EPI-Suite (v4.11) provided by US EPA^12^ and predicts the Henry’s law constants using two-dimensional structure-based quantitative structure/property relationships (QSPRs) calibrated on experimental data.^29^ HenryWin offers the bond contribution method and the group contribution method, but the former was mainly considered here because the latter did not calculate a prediction for any of the eight sulfonamides used in this work. SMILES strings as shown in Table S2 were entered, and the output Henry’s law constants at 25 °C were converted to *K*_aw_ following the ideal gas law.

#### 2.4.2 OPERA

OPERA (v 2.9, downloaded in January 2023) stands for OPEn structure–activity/property Relationship App and is provided by US EPA.^30^ It predicts physicochemical properties based on a *k*-nearest neighbor method using PaDEL descriptors. For *K*_aw_, the model is calibrated on the PhysProp experimental database. As for HenryWin, SMILES strings were the input, and predicted Henry’s law constants at 25 °C were converted to *K*_aw_. For evaluation of the applicability domain (AD), the OPERA software provides a “global AD” based on the leverage calculation and a “local AD index” derived from the similarity to the five nearest neighbor chemicals.^30^

#### 2.4.3 LSER-IFSQSAR

The linear solvation energy relationship (LSER) is a polyparameter linear free energy relationship developed by Abraham and is a multiple linear regression model that describes logarithmic partition coefficients using five solute descriptors.^31,32^ The iterative fragment selection quantitative structure–activity relationship (IFSQSAR)^33^ predicts these solute descriptors. Combined with the published LSER equation for log *K*_aw_,^34^ IFSQSAR predicts log *K*_aw_ values based only on the molecular structure. An online toolbox, EAS-E Suite (Ver. 0.96-Beta, released November 2022, accessed in February 2023)^35^ was used for the calculation. As an AD indicator, EAS-E Suite provides an “uncertainty level (UL)”, which indicates reliability of the predicted solute descriptors. In addition, EAS-E Suite calculates “estimated errors”, which are estimates of overall prediction errors resulting from uncertainty in both solute descriptors and the LSER equation.^33^

#### 2.4.4 COSMO*therm*

COSMO*therm* is a program that predicts activity-related properties including partition coefficients, following the COSMO-RS theory.^36^ Turbomole, COSMO*confX*, and COSMO*thermX* (COSMO*logic*, Dassault Systèmes, version 2021) were used to perform COSMO*therm* calculations, as described previously.^21^ Briefly, for each compound, an initial structure was entered to COSMO*confX*, which generated and evaluated a number of possible conformers. Quantum chemical calculations were performed with Turbomole to obtain “COSMO files”, which contain the necessary information such as molecular surface polarity. COSMO*thermX* read the generated COSMO files, calculated pairwise interaction energy between molecules, and derived partition coefficients from statistical thermodynamics using the COSMO*therm* algorithm. The parameterization applied was BP_TZVPD_FINE_21. Log *K*_Hxd/w_ and log *K*_aw_ at 25 °C were predicted with this method.

## 3 Results and Discussion

### 3.1 Measurement of *K*_Hxd/w_

*K*_Hxd/w_ values measured in this study are summarized in Table 2.

**Table 2:** Log *K*_Hxd/w_ Values Measured in This Study and Log *K*_aw_ Derived from Eq 1

	log *K*_Hxd/w_						log *K*_aw_	
	batch partition method^a^		shared-headspace method^a^		modified VPR-HS method		eq 1	VPR-HS or VP/S
	mean	95% CI	mean	95% CI	mean	95% CI		
3:1 FTOH	NA		NA		–0.35	[−0.37, −0.32]	–1.89	–2.03^d^
3:3 FTOH	0.52	[0.51, 0.54]	NA		0.32	[0.17, 0.47]	–2.17	–2.04^d^
4:2 FTOH	0.79	[0.77, 0.81]	NA		0.75	[0.70, 0.80]	–1.57	–1.56^d^, −1.30^e^, −1.63^f^, −1.21^g^, −1.52^h^
4:4 FTOH	1.60	[1.58, 1.62]	NA		1.43	[1.09, 1.71]	–1.97	–1.28^d^
6:2 FTOH	2.50	[2.42, 2.59]	2.51	[2.44, 2.58]	NA		–0.64	–1.47^e^, −0.56^f^, 0.37^g^, −0.44^h^
7:1 FTOH	NA		2.70	[2.56, 2.84]	NA		–0.26	
8:2 FTOH	NA		3.75	[3.73, 3.78]	NA		0.11	–1.82^e^, 0.58^f^, 0.31^g^, 0.96^h^
5:2s FTOH	2.00	[1.93, 2.08]	2.04	[1.98, 2.11]	1.55	[1.18, 1.75]	–0.15	
4:2 FTI	4.13^b^	[3.89, 4.36]	4.93	[4.86, 4.99]	NA		1.60	
6:1 FTI	4.34^b^	[3.82, 4.86]	5.66	[5.36, 5.95]	NA		2.28	
6:1 FTI-7H	4.06^b^	[3.72, 4.39]	4.62	[4.55, 4.70]	NA		0.70	
6:2 FTAC	4.22^b^	[3.92, 4.51]	5.43	[5.40, 5.46]	NA		1.27	
4:2 FTMAC	4.02^b^	[3.82, 4.21]	4.65	[4.58, 4.72]	NA		0.62	–0.14^d^
PFBSA	–1.15^c^	[−1.20, −1.10]	NA		NA		–4.89	
PFHxSA	0.39^c^	[0.28, 0.51]	NA		NA		–3.94	
PFOSA	1.80^c^	[1.72, 1.87]	NA		NA		–3.04	
MeFBSA	0.82^c^	[0.80, 0.85]	NA		NA		–2.85	
MeFHxSA	2.25^c^	[2.16, 2.34]	NA		NA		–2.01	
EtFHxSA	2.95^c^	[2.85, 3.04]	NA		NA		–1.58	
MeFBSE	0.49	[0.45, 0.52]	NA		NA		–4.35	
EtFHxSE	2.76	[2.66, 2.87]	NA		NA		–3.02	

a *n* = 5. *T* = 25 °C unless otherwise noted.

b Likely inaccurate because of the presence of hexadecane in water.

c 1 mM HCl was used.

d Measured by the VPR-HS method in this study.

e Ref (7).

f Ref (8).

g Ref (9).

h Ref (12), 23 or 24 °C. CI, confidence interval. NA, not available. Underlined values were used for eq 1.

Log *K*_Hxd/w_ values measured by both batch and shared-headspace methods are available for seven compounds. The two methods resulted in practically identical values for 6:2 and 5:2s FTOHs. However, the log *K*_Hxd/w_ values measured by the batch method were significantly lower than those by the shared-headspace method for the remaining five compounds (i.e., 4:2 FTI, 6:1 FTI, 6:1 FTI-7H, 6:2 FTAC, 4:2 FTMAC), which are all relatively hydrophobic. The batch method-measured values for the five compounds were all ∼4 with wide 95% CIs. It is likely that the water phase equilibrated in the batch method contained invisible microdroplets of hexadecane, which increased the apparent concentration of the water phase and decreased the measured (apparent) *K*_Hxd/w_. Indeed, if we assume that the water phase contained 0.006 vol % hexadecane droplets and that log *K*_Hxd/w_ determined by the shared-headspace method is accurate, we can reproduce the “apparent log *K*_Hxd/w_” values measured by the batch method (see SI-4 for the calculation). The presence of 0.006 vol % hexadecane in water suggests that the current batch partition method is applicable to measure log *K*_Hxd/w_ values up to 3.6. For higher *K*_Hxd/w_, an error of >0.1 log unit would occur. The method could be improved by making an additional effort to remove hexadecane from water, e.g., by centrifugation or filtration. In the shared-headspace method, the water phase is free of hexadecane. The shared-headspace method appears applicable for compounds with log *K*_Hxd/w_ values higher than 4, although the test compound should be sufficiently volatile and hydrophobic (see the Methods section). Thus, the batch partition and shared-headspace methods are largely complementary and, by combining these two methods, we were able to measure log *K*_Hxd/w_ from −1.15 to 5.66 for PFAS with different molecular structures.

The modified VPR-HS method resulted in log *K*_Hxd/w_ values comparable to those measured by the batch partition method (Table 2). However, the data plot did not always follow the relationship between PA and *V*_Hxd_ expected by eq 2 (Figure S1). As a result, the CIs were large, particularly for 4:4 and 5:2s FTOHs. The reason may be significant adsorption to, e.g., the glass wall, septum, air/water interface, and syringe used for GC injection, which violates the assumption of eq 2. Thus, although the method is by far the simplest, the use of this method may be limited to weakly sorptive compounds. In the following discussion, we consider only the data for 3:1 FTOH determined by the modified VPR-HS method because no other data are available for this compound.

### 3.2 Determination of *K*_aw_

Using eq 1, we obtained log *K*_aw_ values (25 °C) from −4.89 to 2.28, thus over 7 log units (Table 2). Such a wide range would not be achievable by direct measurement methods alone, demonstrating an advantage of the thermodynamic cycle approach used here. Table 2 also shows log *K*_aw_ values determined by direct methods including the standard VPR-HS method in this study (Figure S2) and the literature as well as values derived from VP/S calculation in the literature. The values for 3:1, 3:3, and 4:2 FTOHs from different sources agree well with each other, mostly within 0.1–0.2 log units. However, larger difference was found for 4:4, 6:2, 8:2 FTOHs, and 4:2 FTMAC, which are less water-soluble and less volatile than the former three compounds and are likely more susceptible to experimental artifacts. As shown below, our values from eq 1 are internally consistent and should represent the most reliable set of experimental values available for PFAS. In the course of this work, a new paper appeared that reported *K*_aw_ values for 15 PFAS measured by the standard VPR-HS method.^37^ These data, however, are inconsistent with the available knowledge for *K*_aw_ of PFAS (see SI-5 and Figures S5, S6 for more details) and thus are not further considered here.

### 3.3 PFAS Molecular Structure and Partition Coefficients

From the data obtained in this work, several important relationships between the structure of PFAS and the partition coefficients can be noted.

Fluorotelomer substances (i.e., FTIs, FTACs, FTMACs) favor air over water (log *K*_aw_ > 0), except for FTOHs. Even with a polar functional group such as acrylate or methacrylate, these chemicals readily partition from water to air. In contrast, unsubstituted PFASAs are water-soluble (log *K*_aw_ < −3), even if their ionization in water as discussed above is disregarded. *N*-methyl substitution of PFASAs increases *K*_aw_ by 2 log units, making them substantially less water favoring. Further *N*-hydroxyethyl substitution (e.g., from MeFBSA to MeFBSE) decreases *K*_aw_ back by 1.5 log units, because of the introduction of the polar −OH group.

**Figure 1:**
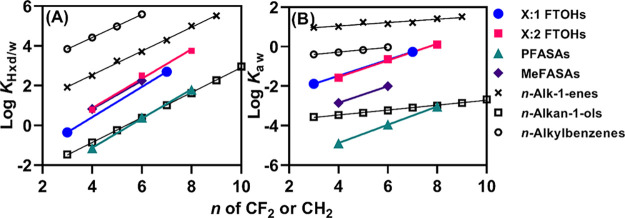
Dependence of (A) log *K*_Hxd/w_ and (B) log *K*_aw_ on the number of CF_2_ or CH_2_ units. Data for *n*-alkan-1-enes, *n*-alkylbenzenes, and *n*-alkan-1-ols are from Abraham et al.^41,42^ The lines indicate the linear regression.

We have *K*_aw_ data for two X:1 FTOHs, three X:2 FTOHs, three PFASAs, and two *N*-methyl FASAs, from which we can calculate the dependence of log *K*_aw_ on the CF_2_ chain length (Figure 1B and Table S4). Log *K*_aw_ of these four groups increases consistently by 0.43 ± 0.02 log units per CF_2_. This value is useful when the log *K*_aw_ for PFAS needs to be extrapolated from available data for shorter-chain homologues. The high consistency of the observed CF_2_ dependence over four classes of PFASs supports accuracy of our data. Noteworthy is that log *K*_aw_ increases only by 0.14 ± 0.04 log units per CH_2_. This small change in log *K*_aw_ with the addition of CH_2_ can be explained by the fact that the increased van der Waals interaction energy with water molecules is nearly offset by the increased cavity formation energy cost. In the case of CF_2_, the increase in cavity formation energy exceeds the increase in interaction energy, and thus, CF_2_ has a substantially larger influence on log *K*_aw_ than CH_2_. Interestingly, the CF_2_ and CH_2_ increments have similar influences on log *K*_Hxd/w_ (0.74 ± 0.02 and 0.64 ± 0.04, respectively; Figure 1A), as opposed to the differing influences on log *K*_aw_.

Another finding is that log *K*_Hxd/w_ of 6:1 FTI-7H is 1.0 log unit lower than that of 6:1 FTI and that log *K*_aw_ of the former is 1.6 log units lower than that of the latter. Both indicate that 6:1 FTI-7H has substantially higher water affinity than 6:1 FTI does. The only structural difference between 6:1 FTI-7H and 6:1 FTI is the F substitution pattern of the terminal methyl group (−CF_2_H vs −CF_3_, respectively). The H atom at the end of the perfluoroalkyl chain can serve as a relatively strong H-bond donor site because of the strong electron-withdrawing property of the F atoms, substantially strengthening the polar interactions with water molecules. It has been reported in the literature that H atoms of halogenated aliphatic compounds can serve as H-bond donor sites (e.g., chloroform,^38^ hexachlorocyclohexanes,^39^ and chlorinated paraffins)^40^ and affect the partition properties of the compounds.

### 3.4 Evaluation of Prediction Models

**Figure 2:**
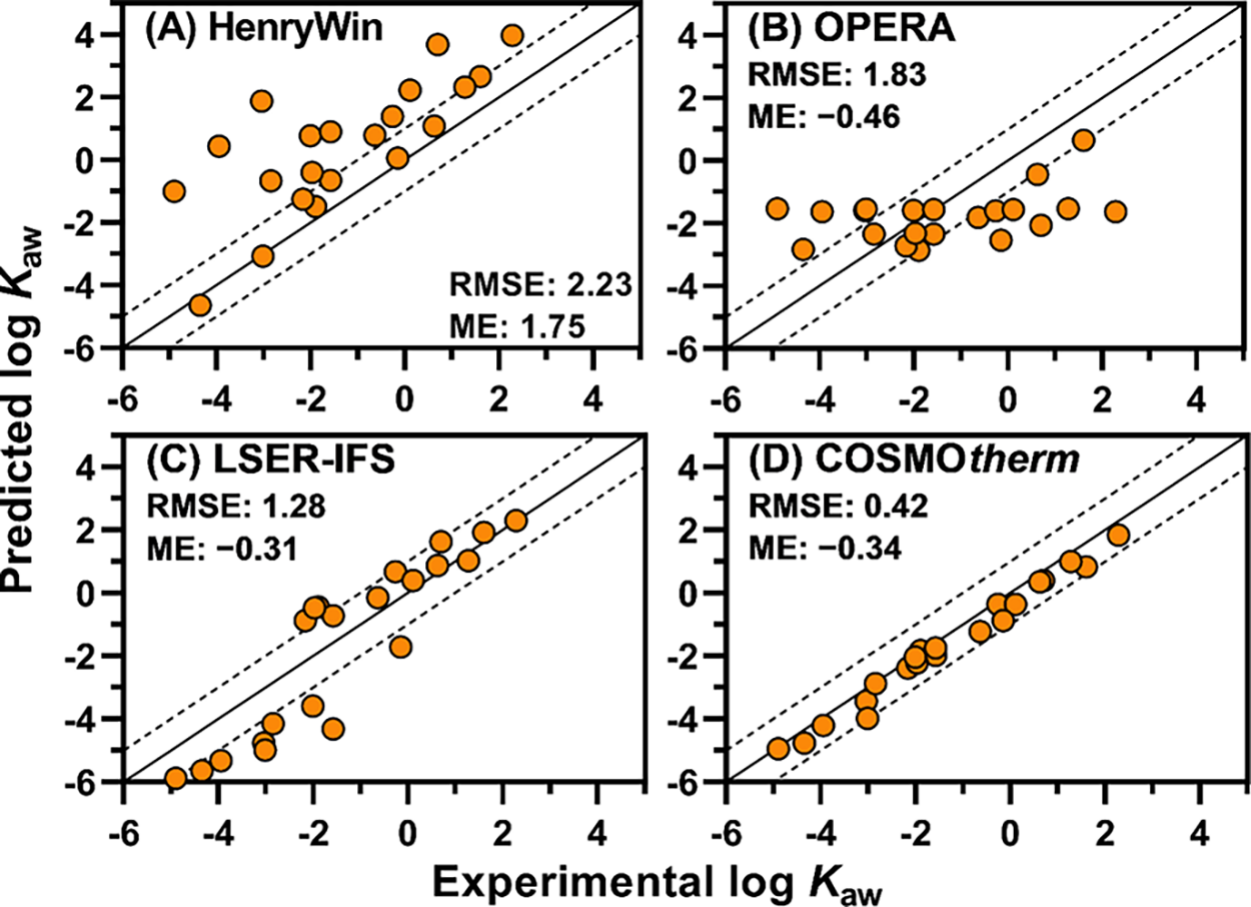
Predicted vs experimental log *K*_aw_ for 21 PFAS. Solid lines indicate the 1:1 agreement and dashed lines 1 log unit difference. A larger version is available in the SI. RMSE, root-mean-squared error; ME, mean error, i.e., the mean of predicted minus experimental values.

Model predictions of log *K*_aw_ are presented in Table S5 and were compared to the experimental values obtained in this study (Figure 2; a larger version is provided in Figure S3). For each model, the root-mean-squared error (RMSE) was calculated from the difference between experimental and predicted values.

HenryWin (bond contribution model) suffered from a high RMSE (2.2 log units) with strong biases to overprediction. Deviations from experimental values increased with increasing chain length. Particularly large errors were found for PFOSA (error, +4.9), PFHxSA (error, +4.4), PFBSA (error, +3.9), and 6:1 FTI-7H (error, +3.0), with the plus sign in the parentheses indicating overprediction. Predictions for FTOHs were better but still errors up to +2.1 were found. The group contribution model of HenryWin was not any better (RMSE 2.1 log units; *n* = 13; see Table S5). In 2006, Arp et al.^20^ demonstrated similar overprediction for four FTOHs by a former version of HenryWin. There appears to be no major improvement in predicting log *K*_aw_ for PFAS.

OPERA also resulted in a high RMSE (1.8 log units). The OPERA prediction did not even follow the increasing trend of log *K*_aw_ with the chain length, but instead, it calculated a narrow range of log *K*_aw_ from −2.9 to −1.5 for 19 out of 21 compounds. More specifically, OPERA calculated ∼ −1.6 for 10 and ∼ −2.3 for 3 compounds. As shown in Table S6, all predictions were outside the global AD defined by the leverage calculation of OPERA. Moreover, the local AD indices generated by OPERA were low (<0.4) for all but 3:1 FTOH, meaning that the five nearest neighbors were not similar to the predicted compound. Thus, OPERA clearly indicated that its log *K*_aw_ predictions for the 21 PFAS were not trustworthy. Looking closer at the output file, we found that a few calibration chemicals (e.g., 6:2, 8:2 FTOHs) were frequently identified as neighboring compounds, which resulted in similar predictions for many of the tested PFAS. Lampic and Parnis^13^ proposed to compute *K*_ow_ and *K*_oa_ with OPERA and apply a thermodynamic cycle to calculate *K*_aw_. This approach, however, did not result in major improvement in RMSE (1.7 log units, Table S5) even though all 21 compounds were considered within AD of both *K*_ow_ and *K*_oa_ prediction models.

LSER-IFSQSAR provided a better RMSE (1.3 log units) than the former two methods, and it captured the chain-length dependence of log *K*_aw_. LSER-IFSQSAR consistently underestimated sulfonamides and their derivatives (errors, −2.7 to −1.0) and tended to overestimate the others including FTOHs (errors, −0.2 to +1.5), except 5:2s FTOH (error, −1.6). The ULs provided by EAS-E Suite did not have a relationship with the observed absolute errors (*R*^2^, 0.01) but the estimated errors from EAS-E Suite did have a positive correlation with the observed absolute errors (*R*^2^, 0.38) (Table S6).

COSMO*therm* was by far the most accurate of the four models tested (RMSE, 0.4 log units). The accuracy of log *K*_aw_ prediction for neutral PFAS is comparable to that for nonfluorinated chemicals (∼0.3 log units).^43^ Thus, COSMO*therm* accurately modeled the influence of the perfluoroalkyl structure on log *K*_aw_ discussed in the previous section. There was slight overall underprediction with a mean error (ME) of −0.34 log units. This minor but consistent bias is similar to the prediction for log *K*_Hxd/air_, reported in our previous paper.^21^ The ME of log *K*_Hxd/air_ predictions for the current 21 compounds is +0.48 (Figure S4). COSMO*therm* appears to slightly overestimate the partitioning of PFAS from air to liquid phases. In contrast, log *K*_Hxd/w_ predictions by COSMO*therm* have little bias (ME, +0.14; Figure S4), suggesting the liquid/liquid partitioning can be predicted more accurately. Note that, if we correct the COSMO*therm*-predicted log *K*_aw_ values by adding 0.34, the RMSE would become 0.26 log units.

Overall, the evaluation of four models presented above suggests that fully empirical models (e.g., HenryWin, OPERA) do not seem to be useful for predicting *K*_aw_ of PFAS, at least for the moment. Errors by 4 or 5 orders of magnitude may be too high even for chemical screening purposes. The low performance might be no surprise, as there have not been sufficient calibration data for PFAS. Apparently, HenryWin lacks fragment values and correction factors required to describe the influence of F substitutions on log *K*_aw_. OPERA needs far more *K*_aw_ data for PFAS that could serve as useful nearest neighbors. HenryWin and OPERA could be recalibrated with the experimental log *K*_aw_ data from this study, which should improve their prediction for PFAS that are similar to any of the test compounds of this study. However, large errors could still occur for the other PFAS. As the architecture of LSER-IFSQSAR is more complicated than that of HenryWin and OPERA, it is not straightforward to interpret the results. LSER-IFSQSAR has been built on the mechanically based LSER equation and calibrated not only on log *K*_aw_ but on many different partition data available, which could explain the better predictions. The high prediction performance of COSMO*therm* indicates that this theoretically based model is suitable for predicting *K*_aw_ of PFAS. Arguably, for an extremely data-poor class of chemicals such as PFAS, a theoretical model that does not require substructure-specific calibration generates more accurate predictions than empirical models. An obstacle to using COSMO*therm* for a broad range of PFAS is that it is not a free tool. For practical and regulatory use, we predicted log *K*_aw_ using COSMO*therm* for 222 PFAS, including 107 neutral PFAS that Kissel et al. recently listed as candidate compounds as well as neutral species of PFAS acids of major concern such as PFOA and its alternatives (Table S7). The predicted log *K*_aw_ values were in the range of −10.0 to 9.7, and 200 (90%) of the 222 values were from −5 to 5, indicating a large variation of the partition properties. Further model validation would be desirable though, considering the diverse structures of existing PFAS. Nevertheless, as long as we do not have reliable experimental data, these predictions may be considered current best estimates and useful for various purposes, such as fate, exposure, and risk analysis.

## Supporting Information Available

Additional descriptions of experimental methods; evaluation of new literature data; lists of all PFAS used; SMILES strings for PFAS; experimental parameters; statistics of the regression lines; all predicted log *K*_aw_ values; figures for experimental methods and raw data; and COSMO*therm*-predicted vs experimental values (PDF)
