# Supplementary material for: Experimental Determination of Air/Water Partition Coefficients for 21 Per- and Polyfluoroalkyl Substances Reveals Variable Performance of Property Prediction Models
Source: Environ Sci Technol. 2023 May 26;57(22):8406–13. doi: 10.1021/acs.est.3c02545 (PMC10249623; doi:10.1021/acs.est.3c02545)
Supplement: Supplementary file 1 — es3c02545_si_001.pdf [file es3c02545_si_001.pdf]

Supporting Information for "Experimental Determination of Air/water Partition Coefficients for 21 Per- and Polyfluoroalkyl Substances (PFAS) Reveals Variable Performance of Property Prediction Models"

Satoshi Endo, Jort Hammer, Sadao Matsuzawa*

Health and Environmental Risk Division, National Institute for Environmental Studies (NIES), Onogawa 16-2, 305-8506 Tsukuba, Ibaraki, Japan

*Corresponding author
Satoshi Endo: phone: +81-29-850-2695, email: endo.satoshi@nies.go.jp

Summary: 27 pages, 5 text sections, 7 tables, 5 figures.

Contents

SI-1 Extended descriptions for experimental methods
SI-2 Conditions for LC/MS and GC/MS analysis
SI-3 Direct measurement of K_{aw} using VPR-HS method
SI-4 Possible presence of hexadecane in water samples of the batch partitioning experiment
SI-5 Evaluation of new K_{aw} data in the literature
Figure SI-5-1. Dependence of log K_{aw} on the number of CF_2.
Figure SI-5-2. Predicted vs experimental log K_{aw}.
Table S1. List of PFAS used in this study with their providers and purity.
Table S2. SMILES strings for PFAS used in this study.
Table S3. Experimental parameters for batch partition and shared-headspace methods.
Table S4. Statistics of the regression lines in Figure 1.
Table S5. Log K_{aw} values obtained via eq 1 and predicted by various models.
Table S6. Indicators of applicability domains (AD) provided by OPERA and LSER-IFSQSAR.
Table S7. Log K_{aw} values of neutral (or neutral species of) PFAS predicted by COSMO*therm*.
Figure S1. Schematic illustrations of experimental methods for determination of partition coefficients.
Figure S2. The results of log $K_{Hxd/w}$ determination by the modified VPR-HS method.
Figure S3. The results of log K_{aw} determination by the standard VPR-HS method.
Figure S4. Predicted vs experimental log K_{aw} (enlarged version of Figure 2 in the main manuscript).
Figure S5. COSMO*therm*-predicted vs experimental values for log $K_{Hxd/w}$, log K_{aw}, and log $K_{Hxd/air}$.
References

SI-1 Extended descriptions for experimental methods

Batch partition method

Pure PFAS were dissolved in hexadecane either directly or using acetone as carrier solvent. Up to three PFAS were mixed in hexadecane and investigated simultaneously. The PFAS concentration in hexadecane was 0.1–2000 mg/L, depending on the expected $K_{Hxd/w}$ and the sensitivity of the quantification method. The acetone content in hexadecane was up to 0.3% (v/v). This acetone should have largely partitioned into water during the partition experiment, where the final concentration in water was estimated to be < 0.3% (v/v). We assumed that this concentration of acetone in water does not have a significant influence on partition coefficients of PFAS.

Batch partition experiments were performed in 10-mL glass vials, which received water and the hexadecane solution of PFAS. The volume of water (V_w) was 1–5 mL, and that of hexadecane was 10 mL – V_w. Five replicate vials were prepared. The vials were crimp-sealed with PTFE-lined septa and were gently shaken for 24 h in an incubation chamber (60 rpm, 25 °C) while kept upright without breaking the hexadecane/water interface. The vials were then collected, flipped upside down slowly so that the water phase came into contact with the septum and kept standing for at least 30 min. An aliquot of the water phase was withdrawn through the septum using a Hamilton syringe. The vials were flipped back, the septum was removed, and the hexadecane phase was taken with a pipette. The water phase was analyzed directly by liquid chromatography/mass spectrometry (LC/MS) (for PFBSA, PFHxSA, MeFBSA, MeFHxSA, MeFBSE), diluted with acetonitrile and analyzed with LC/MS (for PFOSA EtFHxSA, EtFHxSE), or liquid-liquid-extracted with *n*-hexane and analyzed by gas chromatography/mass spectrometry (GC/MS) (for all others). The hexadecane phase was back-extracted with water (for PFBSA, PFHxSA) or with 0.1 mM NaOH water (for PFOSA) and measured by LC/MS, or it was diluted with *n*-hexane by a factor of 100–500 and analyzed by GC/MS (for all others). $K_{Hxd/w}$ was calculated using the measured water phase concentration under the mass conservation assumption. This assumption was confirmed by the hexadecane analysis, which demonstrated 93–112% of the expected concentrations in hexadecane. Note that back-extraction for PFBSA, PFHxSA, and PFOSA was not complete and was corrected for the extraction efficiency determined in a separate batch test. The 95% confidence interval (CI) of the mean (x) was calculated with the formula, $x \pm 2.78$ SD/$\sqrt{5}$, where SD is the standard deviation, based on the t-distribution and n = 5. The PFAS concentrations prepared in hexadecane, water and hexadecane volumes, and recovery from the hexadecane phase for each PFAS are given in Table S2. Note that, as stated in the main text, PFBSA, PFHxSA, PFOSA, MeFBSA, MeFHxSA, and EtFHxSA were performed using 1 mM HCl aqueous solution instead of pure water.

Shared-headspace method

A shared headspace method was used to measure $K_{Hxd/w}$ for nine PFAS. In this method, hexadecane solution of PFAS was prepared as described above and 300 μL was placed in a 350-μL glass insert

accommodated by a 1.5-mL GC vial. This small vial was kept open and put in a 10-mL headspace vial that contains 1 mL of water. The 10-mL vial was closed with a PTFE-lined septum and gently shaken at 25 °C for 24 h. During this equilibration time, the PFAS dissolved in hexadecane was partially transferred to water via the headspace until three-phase partition equilibrium was reached between hexadecane, air, and water. The 10 mL vial was then opened, and water was sampled with a glass pipette and immediately extracted with *n*-hexane for GC/MS analysis. The hexadecane phase was also sampled, diluted with *n*-hexane, and measured with GC/MS, which confirmed 85–100% mass conservation (Table S2). $K_{Hxd/w}$ was obtained from the measured concentration in water, as done in the batch partition method.

Modified VPR-HS method

$K_{Hxd/w}$ was also measured with a modified version of VPR-HS method; 20 mL headspace vials were filled with water and hexadecane with a total liquid volume of 20 mL, leaving a headspace volume of 2.2 mL. All vials received 5 μL of acetonic stock solution (5 or 10 g/L). The vials were shaken standing for 22 h at a room temperature and placed in a GC-sample tray (Tray Cooler 2, Gerstel) controlled at 25.0 °C for 2 h. The headspace was sampled with a syringe using an autosampler and injected into the GC/MS. GC/MS conditions for the headspace measurement have been described previously.[1]

SI-2 Conditions for LC/MS and GC/MS analysis

LC/MS analysis

The LC/MS system consisted of a 1260 Infinity II LC and a single quadrupole MS detector (Agilent Technologies). For analysis of PFAS, 2 µL of each sample solution was injected to the LC/MS equipped with a C_{18} column (Kinetex 2.6 µm EVO C18, 50 × 2.1 mm i.d., Phenomenex) using the autosampler. Separation was performed by applying a gradient elution with a mixture of methanol (A) and water containing 10 mM ammonium formate (B). The solvent volume ratio (A/B) was 40/60 for 0–3 min, linearly changed to 95/5 for 3–6 min, held at 95/5 up to 11 min, returned to 40/60 for 11–11.2 min, and held until end of measurement. The flow rate was 0.25 mL/min for the first 6 min, increased linearly to 0.4 mL/min for 6–10.5 min, held for 10.5–11.2 min, decreased back to 0.25 mL/min, and held until end of the method. The column oven temperature was either 35 or 40 °C. The MSD conditions were as follows: Gas temperature, 350 °C; drying gas flow, 12 L/min; nebulizer pressure, 40 psig; quadrupole temperature 100 °C; capillary voltage, 3000 V; fragmentor voltage, 50 V. Nitrogen gas was generated using an AT-10NP-CS nitrogen generator (Airtech Corporation, Yokohama, Japan). MS chromatograms were obtained using selected ion monitoring (SIM) with the electrospray ionization (ESI) negative mode. The monitored ions for quantifying PFBSA, PFHxSA, PFOSA, MeFBSA, MeFHxSA, EtFHxSA, MeFBSE, and EtFHxSE were m/z 298, 398, 498, 312, 412, 426, 402, and 516, respectively. The chromatographic areas of the samples were compared to those of external standard solutions for quantification.

GC/MS analysis

For GC/MS analysis, a 7890A/5975C GC/MS system (Agilent Technologies), an MPS2 autosampler (Gerstel), and a CIS4 septumless programmed temperature vaporizer (PTV)-type injector (Gerstel) were used. An Rtx-624 column (0.25 mm × 60 m, film thickness 1.4 µm, Restek) was used for separation. Temperature of the GC transfer line was set to 230 °C. Helium was used as carrier gas with a flow rate of 1.2 mL/min. The oven temperature program varied, depending on the chemical properties. A generic program was as following: 50 °C for 1 min, raised to 110 °C at a rate of 30 °C/min, to 190 °C at a rate of 10 °C/min, and to 230 °C at a rate of 30 °C/min, and held for 4 min. The injection volume was 1 or 2 µL. The injector was in the cold splitless injection mode, where the injector temperature was initially 50 °C and was raised immediately after injection to 200 °C at a rate of 12 °C/s. The hexadecane peak usually appeared after all target PFAS had been eluted. The MS conditions were as following: ion source temperature, 230 °C; quadrupole temperature, 150 °C; gain factor, 1. The selected ion monitoring (SIM) mode was used for quantification. Two or three major ions were monitored for each PFAS. For quantification of the *n*-hexane extracts of water samples, internal standards (IS) were added to the extracts. The PFAS was quantified relative to the IS peak area. The compounds used as IS were: 4:2 FTOH for 5:2s and 6:2 FTOHs; 5:2s FTOH for 3:3, 4:2, and 4:4 FTOHs; 6:2 FTOH for 7:1 and 8:2 FTOHs; 6:2 FTI for 4:2 FTI, 6:1 FTI, 6:1 FTI-7H, 6:2 FTAC, and 4:2 FTMAC. For

hexadecane samples diluted by *n*-hexane, no IS was added, and peak areas of PFAS were directly used for quantification. As shown in Table S2, the repeatability was high even without IS, with a relative standard deviation of 11% in the worst case. A single point calibration was performed as the response linearity was generally high. The concentration of the external standard was greater than the sample concentration but less than 10 times greater.

SI-3 Direct measurement of K_{aw} using VPR-HS method

Pure liquid or solid of PFAS (ca 1 µL or 1 mg) was directly dissolved in 20 or 200 mL of water. The solution was shaken for 24 h in a sealed container and distributed into 15 of 20-mL headspace vials. The volumes of water in the vials were varied in the range of 0.1 to 3 mL or 1 to 16 mL. The headspace vials were closed with PTFE-lined septa, weighed, and equilibrated at 25°C for at least 2 h. The internal volume of the vials was 22.16 ± 0.09 mL, as measured before.[1] The vials were put on a sample tray (Tray Cooler 2, Gerstel) where the temperature was controlled at 25 °C. The headspace was then sampled and measured by using the GC/MS system (7890A GC/5975C MS, Agilent Technologies; MPS2 autosampler, Gerstel). The analytical conditions of GC/MS were the same as described in ref 1. Under the mass conservation assumption, the measured GC peak area (PA) can be expressed as eq S1,[2-4]

$$PA = \frac{rC_{\mathrm{w}}}{\frac{1}{K_{\mathrm{aw}}}+\frac{V_{\mathrm{HS}}}{V_{\mathrm{w}}}} \qquad \text{(S1)}$$

where r is the response factor between PA and the concentration of the analyte in the headspace, C_{w} is the initial concentration of the analyte in water, and $V_{\mathrm{HS}}/V_{\mathrm{w}}$ is the headspace-to-water volume ratio. The equation was fitted to the data by adjusting rC_{w} and K_{aw} using GraphPad Prism 9.5 with weighting factors ($1/y^2$). For more details about data evaluation, see ref 1.

SI-4 Possible presence of hexadecane in water samples of the batch partitioning experiment

The batch partition method-measured log $K_{Hxd/w}$ values for five compounds (i.e., 4:2 FTI, 6:1 FTI, 6:1 FTI-7H, 6:2 FTAC, 4:2 FTMAC) were all ~4, which may be explained by possible hexadecane microdroplets in water. If hexadecane is present in the water sample, the apparent aqueous phase concentration of the chemical ($C_{w,app}$) can be expressed as the sum of the contributions from water and hexadecane to the total concentration; thus,

$$C_{w,app} = C_w (\varphi_w + \varphi_{Hxd} K_{Hxd/w}) \quad \text{(S2)}$$

where C_w is the true aqueous phase concentration of the chemical and φ_w and φ_{Hxd} are the volume fractions of water and hexadecane, respectively, in the water sample. The corresponding apparent $K_{Hxd/w}$ measured in the batch partition method ($K_{Hxd/w,app}$) is,

$$K_{Hxd/w,app} = C_{Hxd}/[C_w (\varphi_w + \varphi_{Hxd} K_{Hxd/w})] \quad \text{(S3)}$$

where C_{Hxd} is the chemical concentration in hexadecane. Since $C_{Hxd}/C_w = K_{Hxd/w}$ and φ_w ~ 1, eq S3 can be rewritten as,

$$K_{Hxd/w,app} = K_{Hxd/w}/(1 + \varphi_{Hxd} K_{Hxd/w}) \quad \text{(S4)}$$

By rearranging eq S4, we obtain,

$$\varphi_{Hxd} = 1/K_{Hxd/w,app} - 1/K_{Hxd/w} \quad \text{(S5)}$$

By inserting the $K_{Hxd/w,app}$ value measured by the batch partition method and the $K_{Hxd/w}$ value measured by the shared-headspace method in eq S5, we can obtain the value of φ_{Hxd}. The values of φ_{Hxd} obtained this way for 4:2 FTI, 6:1 FTI, 6:1 FTI-7H, 6:2 FTAC, and 4:2 FTMAC were 0.000043 to 0.000073, with the mean of 0.000060 (or 0.0060%).

SI-5 Evaluation of new K_{aw} data in the literature

In the course of this work, a new paper appeared that reported K_{aw} values for 15 PFAS measured by the VPR-HS method.[5] These data, however, are inconsistent with the available knowledge for K_{aw} of PFAS, as explained below.

Abusallout et al.[5] measured the dimensionless Henry's law constant k_H, which is identical to K_{aw} denoted in this work, for 4 FTOHs, 3 fluorotelomer sulfonic acids (FTSs), pefluorohexane iodide (PFHxI), 1H,2H-perfluoro-1-iodooct-1-ene (6:2 FTUI), 6:2 FTI, EtFOSA, MeFOSA, 6:2 fluorotelomer olefin (6:2 FTO), 8:2 fluorotelomer carboxylic acid (8:2 FTCA), and 8:2 fluorotelomer acrylate (8:2 FTAC) at 25 °C using Milli-Q water (i.e., without pH control). The measured pH values were 5.7–7.0. In Figure S5, selected experimental data of log K_{aw} from this study and refs 5 and 6 are plotted against the number of CF_2 units to check the data consistency. As discussed in the manuscript, the data from this study showed consistent slopes (0.43 ± 0.02) across different classes of PFAS. The data from Goss et al.[6] also showed a comparable slope (0.53 ± 0.03). The data from ref 5, however, indicated a slope of 0.20 ± 0.06 (FTOHs) and 0.08 ± 0.03 (FTSs), significantly smaller than those of the former two studies. As discussed by Goss et al.,[6] such a gentle slope is unlikely considering the hydrophobic nature of the perfluoroalkyl structure. Figure S6 compares the data from ref 5 to predictions by COSMO*therm* computed in this study. For comparison, experimental data from this study are also included in the plot. Many data from ref 5 strongly deviate from COSMO*therm* predictions. Errors of many orders of magnitude in COSMO*therm* predictions are unlikely for such simply structured PFAS, considering the good agreement of predictions and experimental data from the current study. It should also be noted that the data from ref 5 include 4 acids (3 FTSs and 8:2 FTCA), which dissociate and become ionic in pure water. Thus, the apparent K_{aw} values measured around neutral pH (which are displayed in Figure S6) should be substantially lower than the actual K_{aw} values for the neutral species. However, the apparent K_{aw} data from ref 5 are *higher* than the COSMO*therm* values for the neutral species by up to 5.5 log units. Additionally, it is inconsistent that the log K_{aw} values for 6:2 FTI (−0.52) and 8:2 FTAC (−0.50) from ref 5 are lower than the experimental values for 4:2 FTI (1.60) and 6:2 FTAC (1.27), respectively, from this study, because it is generally expected that log K_{aw} of a longer compound is higher than that of a shorter analogue, as shown in Figure S5. For these reasons, we believe that the data from ref 5 are substantially influenced by experimental artifacts, e.g., sorption to the glass surface and air/water interface.

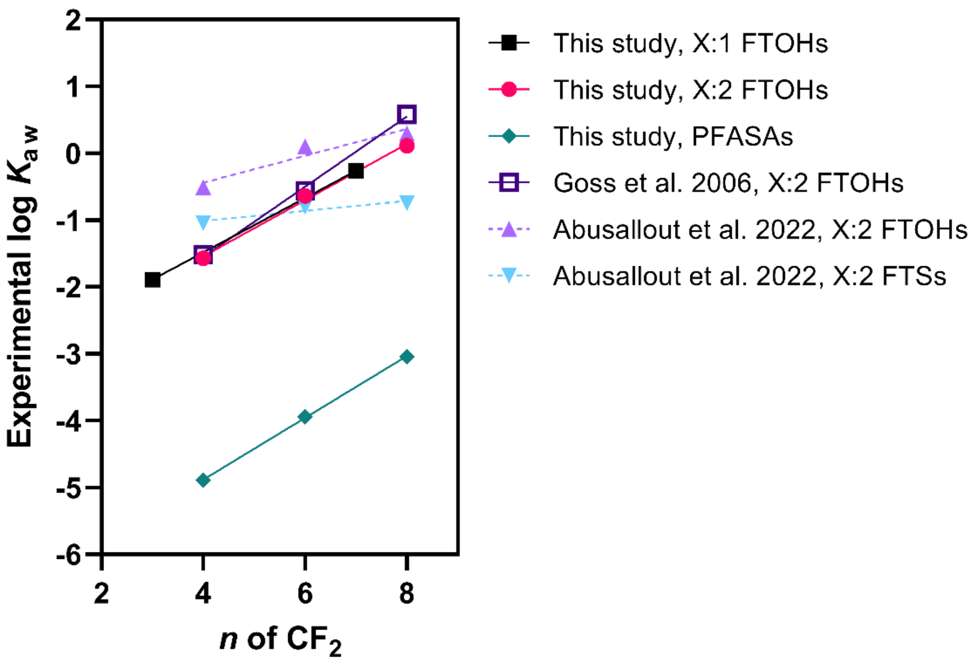

Figure SI-5-1. Dependence of log K_{aw} on the number of CF_2.

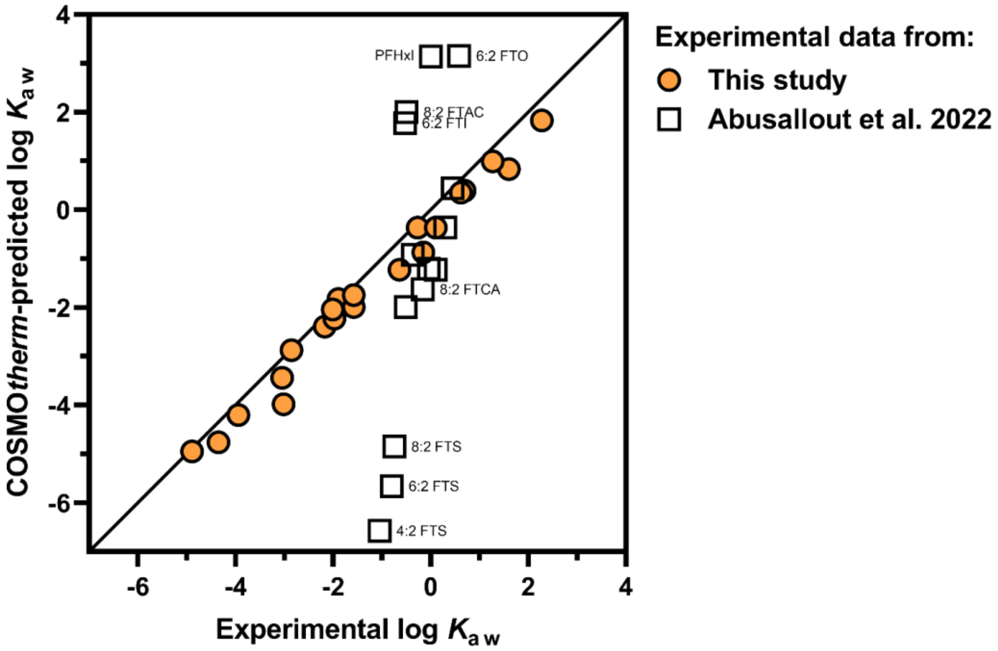

Figure SI-5-2. Predicted vs experimental log K_{aw}. Predicted data are all from this study.

Table S1. List of PFAS used in this study with their providers and purity.

Name	Abbreviation	CAS-RN	Group	Provider	Purity
1H,1H-Perfluorobutan-1-ol	3:1 FTOH	375-01-9	FTOHs	TCI	98
3-(Perfluoropropyl)propan-1-ol	3:3 FTOH	679-02-7	FTOHs	Apollo Scientific	95
1H,1H,2H,2H-Perfluorohexan-1-ol	4:2 FTOH	2043-47-2	FTOHs	TCI	97
4-(Perfluorobutyl)butan-1-ol	4:4 FTOH	3792-02-7	FTOHs	SynQuest	98
1H,1H,2H,2H-Perfluorooctan-1-ol	6:2 FTOH	647-42-7	FTOHs	TCI	98
1H,1H-Perfluorooctan-1-ol	7:1 FTOH	307-30-2	FTOHs	SynQuest	98
1H,1H,2H,2H-Perfluorodecan-1-ol	8:2 FTOH	678-39-7	FTOHs	TCI	97
1H,1H,1H,2H-Perfluoroheptan-2-ol	5:2s FTOH	914637-05-1	FTOHs	SynQuest	97
1H,1H,2H,2H-Perfluorohexyl iodide	4:2 FTI	2043-55-2	FTIs	TCI	99
1H,1H-Perfluoroheptyl iodide	6:1 FTI	212563-43-4	FTIs	Fujifilm-Wako	97
1H,1H,7H-Perfluoroheptyl iodide	6:1 FTI-7H	376-32-9	FTIs	Fluorochem	98
1H,1H,2H,2H-Perfluorooctyl acrylate	6:2 FTAC	17527-29-6	FTACs	TCI	98
1H,1H,2H,2H-Perfluorohexyl methacrylate	4:2 FTMAC	1799-84-4	FTMACs	TCI	98
Perfluorobutane sulfonamide	PFBSA	30334-69-1	PFASAs	SynQuest	97
Perfluorohexane sulfonamide	PFHxSA	41997-13-1	PFASAs	SynQuest	95 (sum of isomers)
Perfluorooctane sulfonamide	PFOSA	754-91-6	PFASAs	SynQuest	85 (sum of isomers)
N-Methyl perfluorobutane sulfonamide	MeFBSA	68298-12-4	FASAs	SynQuest	97
N-Methyl perfluorohexane sulfonamide	MeFHxSA	68259-15-4	FASAs	SynQuest	97
N-Ethyl perfluorohexane sulfonamide	EtFHxSA	87988-56-5	FASAs	SynQuest	97
N-Methyl perfluorobutane sulfonamidoethanol	MeFBSE	34454-97-2	FASEs	TRC	97
N-Ethyl perfluorohexane sulfonamidoethanol	EtFHxSE	34455-03-3	FASEs	SynQuest	99

Table S2. SMILES strings for PFAS used in this study.

3:1 FTOH	C(C(C(C(F)(F)F)(F)F)(F)F)O
3:3 FTOH	C(CC(C(C(F)(F)F)(F)F)(F)F)CO
4:2 FTOH	FC(F)(C(F)(F)CCO)C(F)(F)C(F)(F)F
4:4 FTOH	C(CCO)CC(C(C(C(F)(F)F)(F)F)(F)F)(F)F
6:2 FTOH	C(CO)C(C(C(C(C(C(F)(F)F)(F)F)(F)F)(F)F)(F)F)(F)F
7:1 FTOH	C(C(C(C(C(C(C(C(F)(F)F)(F)F)(F)F)(F)F)(F)F)(F)F)(F)F)O
8:2 FTOH	C(CO)C(C(C(C(C(C(C(C(F)(F)F)(F)F)(F)F)(F)F)(F)F)(F)F)(F)F)(F)F
5:2s FTOH	CC(C(C(C(C(C(F)(F)F)(F)F)(F)F)(F)F)(F)F)O
4:2 FTI	C(CI)C(C(C(C(F)(F)F)(F)F)(F)F)(F)F
6:1 FTI	C(C(C(C(C(C(C(F)(F)F)(F)F)(F)F)(F)F)(F)F)(F)F)I
6:1 FTI-7H	C(C(C(C(C(C(C(F)F)(F)F)(F)F)(F)F)(F)F)(F)F)I
6:2 FTAC	C=CC(=O)OCCC(C(C(C(C(C(F)(F)F)(F)F)(F)F)(F)F)(F)F)(F)F
4:2 FTMAC	CC(=C)C(=O)OCCC(C(C(C(F)(F)F)(F)F)(F)F)(F)F
PFBSA	FC(F)(C(F)(F)S(N)(=O)=O)C(F)(F)C(F)(F)F
PFHxSA	C(C(C(C(F)(F)S(=O)(=O)N)(F)F)(F)F)(C(C(F)(F)F)(F)F)(F)F
PFOSA	C(C(C(C(C(F)(F)S(=O)(=O)N)(F)F)(F)F)(F)F)(C(C(C(F)(F)F)(F)F)(F)F)(F)F
MeFBSA	CNS(=O)(=O)C(C(C(C(F)(F)F)(F)F)(F)F)(F)F
MeFHxSA	FC(F)(C(F)(F)C(F)(F)C(F)(F)C(F)(F)C(F)(F)F)S(=O)(=O)NC
EtFHxSA	FC(F)(C(F)(F)C(F)(F)C(F)(F)C(F)(F)C(F)(F)F)S(=O)(=O)NCC
MeFBSE	CN(CCO)S(=O)(=O)C(C(C(C(F)(F)F)(F)F)(F)F)(F)F
EtFHxSE	FC(F)(C(F)(F)C(F)(F)C(F)(F)C(F)(F)C(F)(F)F)S(=O)(=O)N(CCO)CC

Table S3. Experimental parameters for batch partition and shared-headspace methods.

Single or mixture		Initial conc in hxd mg/L	Liquid volumes in batch partition method mL		Liquid volumes in shared-HS method mL		Water phase composition	Quantification method		Measured conc in hxd (% of expected conc)	
			Water	Hxd	Water	Hxd		Water	Hxd	Batch partition	Shared-HS
Mix A	3:3 FTOH	1	2	8	-	-	pure water	LLE w/ hexane, GC/MS	hexane-dilution, GC/MS	112±8	-
Mix A	4:2 FTOH	1	2	8	-	-	pure water	LLE w/ hexane, GC/MS	hexane-dilution, GC/MS	107±11	-
Mix A	4:4 FTOH	4	2	8	-	-	pure water	LLE w/ hexane, GC/MS	hexane-dilution, GC/MS	NA	-
Mix B	6:2 FTOH	20	5	5	1	0.3	pure water	LLE w/ hexane, GC/MS	hexane-dilution, GC/MS	102±3	89±2
Mix C	7:1 FTOH	50	-	-	1	1.5	pure water	LLE w/ hexane, GC/MS	hexane-dilution, GC/MS	-	99±2
Mix C	8:2 FTOH	500	-	-	1	1.5	pure water	LLE w/ hexane, GC/MS	hexane-dilution, GC/MS	-	99±2
Mix B	5:2s FTOH	10	5	5	1	0.3	pure water	LLE w/ hexane, GC/MS	hexane-dilution, GC/MS	104±2	85±2
Mix D	4:2 FTI	1000	5	5	1	0.3	pure water	LLE w/ hexane, GC/MS	hexane-dilution, GC/MS	97±1	96±2
Mix E	6:1 FTI	2000	5	5	1	0.3	pure water	LLE w/ hexane, GC/MS	hexane-dilution, GC/MS	100±1	97±4
Mix E	6:1 FTI-7H	2000	5	5	1	0.3	pure water	LLE w/ hexane, GC/MS	hexane-dilution, GC/MS	101±1	100±4
Mix D	6:2 FTAC	1000	5	5	1	0.3	pure water	LLE w/ hexane, GC/MS	hexane-dilution, GC/MS	96±2	97±2
Mix D	4:2 FTMAC	1000	5	5	1	0.3	pure water	LLE w/ hexane, GC/MS	hexane-dilution, GC/MS	97±3	97±2
Mix F	PFBSA	0.1	1	9	-	-	1mM HCl	direct LC/MS	water, LC/MS	93±6	-
Mix F	PFHxSA	0.1	1	9	-	-	1mM HCl	direct LC/MS	water, LC/MS	100±8	-
single	PFOSA	1	5	5	-	-	1mM HCl	ACN-dilution, LC/MS	0.1 mM NaOH, LC/MS	94±17	-
Mix G	MeFBSA	1	3	7	-	-	1mM HCl	direct LC/MS	water, LC/MS	100±2	-
Mix G	MeFHxSA	5	3	7	-	-	1mM HCl	direct LC/MS	water, LC/MS	101±3	-
single	EtFHxSA	10	5	5	-	-	1mM HCl	ACN-dilution, LC/MS	hexane-dilution, GC/MS	102±4	-
single	MeFBSE	0.1	2	8	-	-	pure water	direct LC/MS	hexane-dilution, GC/MS	NA	-
single	EtFHxSE	10	5	5	-	-	pure water	ACN-dilution, LC/MS	hexane-dilution, GC/MS	105±5	-

Hxd, hexadecane; NA, not available, LLE, liquid-liquid extraction; ACN, acetonitrile,

Table S4. Statistics of the regression lines in Figure 1.

		X:1 FTOHs	X:2 FTOHs	PFASAs	MeFASAs	*n*-Alk-1-enes	*n*-Alkyl-benzenes	*n*-Alkan-1-ols
Log $K_{Hxd/w}$								
Slope	Value	0.763	0.741	0.737	0.713	0.600	0.581	0.629
	SE		0.066	0.021		0.012	0.007	0.004
Intercept	Value	-2.639	-2.096	-4.074	-2.028	0.135	2.093	-3.375
	SE		0.412	0.128		0.077	0.032	0.030
R^2			0.9921	0.9992		0.9979	0.9997	0.9997
SD			0.187	0.058		0.065	0.015	0.029
n		2	3	3	2	8	6	9
Log K_{aw}								
Slope	Value	0.407	0.421	0.463	0.422	0.086	0.120	0.125
	SE		0.027	0.008		0.013	0.006	0.004
Intercept	Value	-3.109	-3.226	-6.733	-4.535	0.699	-0.760	-3.961
	SE		0.170	0.047		0.081	0.029	0.029
R^2			0.9958	0.9997		0.9004	0.9945	0.9930
SD			0.077	0.021		0.068	0.014	0.028
n		2	3	3	2	8	6	9

Table S5. Log K_{aw} values obtained via eq 1 and predicted by various models.

	Experimental (eq 1)	COSMO*therm*	HenryWin bond	HenryWin group	OPERA direct	OPERA K_{ow}/K_{oa}	LSER-IFSQSAR
3:1 FTOH	-1.89	-1.83	-1.50	-1.32	-2.86	-0.86	-0.45
3:3 FTOH	-2.17	-2.39	-1.25	-1.02	-2.73	-1.72	-0.88
4:2 FTOH	-1.57	-1.99	-0.65	-0.17	-2.34	-0.55	-0.72
4:4 FTOH	-1.97	-2.22	-0.41	0.13	-2.32	-0.01	-0.47
6:2 FTOH	-0.64	-1.23	0.79	1.83	-1.82	0.47	-0.15
7:1 FTOH	-0.26	-0.36	1.38	2.68	-1.61	-0.29	0.67
8:2 FTOH	0.11	-0.36	2.23	3.83	-1.57	1.48	0.40
5:2s FTOH	-0.15	-0.87	0.07	1.05	-2.54	-0.15	-1.71
4:2 FTI	1.60	0.83	2.66	3.01	0.64	-0.22	1.92
6:1 FTI	2.28	1.83	3.97	4.86	-1.63	1.28	2.30
6:1 FTI-7H	0.70	0.39	3.68	3.16	-2.06	0.29	1.63
6:2 FTAC	1.27	1.00	2.32	2.94	-1.53	1.33	1.03
4:2 FTMAC	0.62	0.35	1.08	1.74	-0.44	-0.76	0.87
PFBSA	-4.89	-4.95	-1.01	NA	-1.54	-0.06	-5.88
PFHxSA	-3.94	-4.20	0.44	NA	-1.63	-1.29	-5.32
PFOSA	-3.04	-3.44	1.88	NA	-1.59	-0.85	-4.76
MeFBSA	-2.85	-2.88	-0.66	NA	-2.34	-1.84	-4.15
MeFHxSA	-2.01	-2.04	0.78	NA	-1.59	-1.87	-3.59
EtFHxSA	-1.58	-1.75	0.90	NA	-1.57	-1.41	-4.31
MeFBSE	-4.35	-4.76	-4.64	NA	-2.83	-1.92	-5.65
EtFHxSE	-3.02	-3.98	-3.07	NA	-1.55	-2.30	-4.98
RMSE		0.42	2.23	2.09	1.83	1.67	1.28
ME		-0.34	1.75	1.91	-0.46	0.79	-0.31
SD		0.26	1.43	0.89	1.81	1.51	1.27
n		21	21	13	21	21	21

RMSE, root mean squared error; ME, mean error; SD, standard deviation; NA, not available.

Table S6. Indicators of applicability domains (AD) provided by OPERA and LSER-IFSQSAR.

	OPERA K_{aw}		OPERA K_{ow}			OPERA K_{oa}			LSER-IFSQSAR	
	Global AD	AD index	Global AD	AD index	Confidence index	Global AD	AD index	Confidence index	UL	Error estimate
3:1 FTOH	outside	0.458	inside	1.00	0.95	inside	0.937	0.749	2	1.05
3:3 FTOH	outside	0.177	inside	0.56	0.61	inside	0.972	0.912	2	1.05
4:2 FTOH	outside	0.176	inside	1.00	0.82	inside	0.965	0.898	1	1.04
4:4 FTOH	outside	0.168	inside	0.56	0.56	inside	0.956	0.789	1	1.04
6:2 FTOH	outside	0.175	inside	1.00	0.83	inside	0.965	0.859	1	1.04
7:1 FTOH	outside	0.175	inside	0.61	0.49	inside	0.968	0.913	1	1.04
8:2 FTOH	outside	0.160	inside	1.00	0.90	inside	0.956	0.78	1	1.05
5:2s FTOH	outside	0.177	inside	0.62	0.62	inside	0.966	0.899	0	0.68
4:2 FTI	outside	0.324	inside	0.48	0.22	inside	0.986	0.717	2	0.32
6:1 FTI	outside	0.270	inside	0.51	0.49	inside	0.984	0.866	2	0.33
6:1 FTI-7H	outside	0.274	inside	0.44	0.23	inside	0.986	0.891	1	0.89
6:2 FTAC	outside	0.221	inside	1.00	0.86	inside	0.995	0.934	1	0.51
4:2 FTMAC	outside	0.207	inside	0.55	0.45	inside	0.993	0.929	1	0.51
PFBSA	outside	0.135	inside	0.47	0.68	inside	0.964	0.903	1	0.97
PFHxSA	outside	0.178	inside	0.45	0.27	inside	0.974	0.871	1	0.98
PFOSA	outside	0.155	inside	0.52	0.16	inside	0.964	0.803	1	0.98
MeFBSA	outside	0.135	inside	0.45	0.41	inside	0.976	0.913	2	1.38
MeFHxSA	outside	0.175	inside	0.46	0.25	inside	0.951	0.834	2	1.38
EtFHxSA	outside	0.170	inside	0.47	0.14	inside	0.94	0.764	1	1.08
MeFBSE	outside	0.171	inside	0.49	0.53	inside	0.869	0.735	2	1.54
EtFHxSE	outside	0.157	inside	0.47	0.34	inside	0.831	0.542	2	1.54

UL, uncertainty level.

Table S7. Log K_{aw} values of neutral (or neutral species of) PFAS predicted by COSMO*therm*.

Name	Abbreviation	CAS-RN	log K_{aw} @25°C
1H,1H-Perfluorobutan-1-ol	3:1 FTOH	375-01-9	-1.83
3-(Perfluoropropyl)propan-1-ol	3:3 FTOH	679-02-7	-2.39
1H,1H,2H,2H-Perfluorohexan-1-ol	4:2 FTOH	2043-47-2	-1.99
4-(Perfluorobutyl)butan-1-ol	4:4 FTOH	3792-02-7	-2.22
1H,1H,2H,2H-Perfluorooctan-1-ol	6:2 FTOH	647-42-7	-1.23
1H,1H-Perfluorooctan-1-ol	7:1 FTOH	307-30-2	-0.36
1H,1H,2H,2H-Perfluorodecan-1-ol	8:2 FTOH	678-39-7	-0.36
1H,1H,2H,2H-Perfluorododecan-1-ol	10:2 FTOH	865-86-1	0.44
1H,1H,2H,2H-Perfluorotetradecan-1-ol	12:2 FTOH	39239-77-5	1.26
1H,1H,2H,2H-Perfluorobutan-1-ol	2:2 FTOH	54949-74-5	-2.26
1H,1H,1H,2H-Perfluoroheptan-2-ol	5:2s FTOH	914637-05-1	-0.87
1H,1H,1H,2H-Perfluorononan-2-ol	7:2s FTOH	24015-83-6	-0.13
3-(Perfluoro-2-butyl)propane-1,2-diol	NFHp-1,2-diol	125070-38-4	-4.51
Pentafluoropropanoic anhydride	PFPrAnhy	356-42-3	2.89
Perfluoroheptane	PFHp	335-57-9	4.93
Perfluorooctane	PFO	307-34-6	5.39
Perfluorononane	PFN	375-96-2	5.79
perfluorodecane	PFD	307-45-9	6.23
perfluoroundacane	PFUnD	307-49-3	6.65
Perfluorododecane	PFDoD	307-59-5	7.12
Perfluorobutanesulfonyl fluoride	PFBSF	375-72-4	2.58
Perfluorohexanesulfonyl fluoride	PFHxSF	423-50-7	3.50
Perfluorooctanesulfonyl fluoride	PFOSF	307-35-7	4.43
Perfluorobutyl iodide	PFBI	423-39-2	2.23
Perfluorohexyl iodide	PFHxI	355-43-1	3.14
Perfluoroheptyl iodide	PFHpI	335-58-0	3.57
Perfluorooctyl iodide	PFOI	507-63-1	4.02
Perfluorodecyl iodide	PFDI	423-62-1	4.94
1,8-Diiodoperfluorooctane	1,8-DIPFO	335-70-6	2.62
1H,1H,2H,2H-Perfluorohexyl iodide	4:2 FTI	2043-55-2	0.83
1H,1H-Perfluoroheptyl iodide	6:1 FTI	212563-43-4	1.83
1H,1H,7H-Perfluoroheptyl iodide	6:1 FTI-7H	376-32-9	0.39
1H,1H,2H,2H-Perfluorooctyl iodide	6:2 FTI	2043-57-4	1.78
1H,1H,2H,2H-Perfluorodecyl iodide	8:2 FTI	2043-53-0	2.69
1H,1H,2H,2H-Perfluorododecyl iodide	10:2 FTI	2043-54-1	3.68
1,1,1,2,2,3,3-Heptafluoro-3-[(1,1,1,2,3,3-hexafluoro-3-{[1,1,1,2,3,3-hexafluoro-3-(1,2,2,2-tetrafluoroethoxy)-2-propanyl]oxy}-2-propanyl)oxy]propane	FE-E3	3330-16-3	6.58
1,1,1,2,4,4,5,7,7,8,10,10,11,13,13,14,14,15,15,15-Eicosafluoro-5,8,11-tris(trifluoromethyl)-3,6,9,12-tetraoxapentadecane	FE-E4	26738-51-2	8.24
1,1,1,2,4,4,5,7,7,8,10,10,11,13,13,14,16,16,17,17,18,18,18-Tricosafluoro-5,8,11,14-tetrakis(trifluoromethyl)-3,6,9,12,15-pentaoxaoctadecane	FE-E5	37486-69-4	9.70
Allyl 1H,1H-perfluorooctyl ether	AFOE	812-72-6	2.63
1-(Heptafluoropropoxy)-1,2,2,2-tetrafluoro-1-iodoethane	FE-E1-I	107432-46-2	3.35
Allyl perfluoroisopropyl ether	APFIPE	15242-17-8	1.99

Perfluorotripropyl amine	PFTPrA	338-83-0	5.51
Perfluorotributyl amine	PFTBA	311-89-7	7.00
1H,8H-Perfluorooctane	1,8-DHPFO	307-99-3	2.51
1,8-Divinylperfluorooctane	1,8-DVPFO	35192-44-0	2.68
4-(Perfluorooct-1-yl)styrene	PFOSt	106209-21-6	3.05
1H,1H,2H-Perfluoro-1-hexene	4:2 FTO	19430-93-4	2.32
1H,1H,2H-Perfluoro-1-octene	6:2 FTO	25291-17-2	3.15
1H,1H,2H-Perfluoro-1-decene	8:2 FTO	21652-58-4	4.11
1H,1H,2H-Perfluoro-1-dodecene	10:2 FTO	30389-25-4	4.86
1H,1H,2H,2H-Perfluorohexyl acrylate	4:2 FTAC	52591-27-2	0.14
1H,1H,2H,2H-Perfluorooctyl acrylate	6:2 FTAC	17527-29-6	1.00
1H,1H,2H,2H-Perfluorodecyl acrylate	8:2 FTAC	27905-45-9	2.00
1H,1H,2H,2H-Perfluorododecyl acrylate	10:2 FTAC	17741-60-5	3.04
1H,1H,2H,2H-Perfluorohexyl methacrylate	4:2 FTMAC	1799-84-4	0.35
1H,1H,2H,2H-Perfluorooctyl methacrylate	6:2 FTMAC	2144-53-8	1.07
1H,1H,2H,2H-Perfluorodecyl methacrylate	8:2 FTMAC	1996-88-9	1.79
1H,1H,2H,2H-Perfluorododecyl methacrylate	10:2 FTMAC	2144-54-9	3.08
4-(1H,1H,2H,2H-Perfluorooctyl)benzyl alcohol	6:2 FTBnOH	356055-76-0	-2.54
1H,1H,2H,2H-Perfluorodecyl acetate	8:2 FTAce	37858-04-1	1.37
Perfluorobutane sulfonamide	PFBSA	30334-69-1	-4.95
Perfluorohexane sulfonamide	PFHxSA	41997-13-1	-4.20
Perfluorooctane sulfonamide	PFOSA	754-91-6	-3.44
N-Methyl perfluorobutane sulfonamide	MeFBSA	68298-12-4	-2.88
N-Methyl perfluorohexane sulfonamide	MeFHxSA	68259-15-4	-2.04
N-Methyl perfluorooctane sulfonamide	MeFOSA	31506-32-8	-1.20
N-Ethyl perfluorobutane sulfonamide	EtFBSA	40630-67-9	-3.69
N-Ethyl perfluorohexane sulfonamide	EtFHxSA	87988-56-5	-1.75
N-Ethyl perfluorooctane sulfonamide	EtFOSA	4151-50-2	-0.92
N-Methyl perfluorobutane sulfonamidoethanol	MeFBSE	34454-97-2	-4.76
N-Methyl perfluorohexane sulfonamidoethanol	MeFHxSE	68555-75-9	-3.97
N-Methyl perfluorooctane sulfonamidoethanol	MeFOSE	24448-09-7	-3.11
N-Ethyl perfluorobutane sulfonamidoethanol	EtFBSE	34449-89-3	-4.43
N-Ethyl perfluorohexane sulfonamidoethanol	EtFHxSE	34455-03-3	-3.98
N-Ethyl perfluorooctane sulfonamidoethanol	EtFOSE	1691-99-2	-3.15
N,N-Dimethyl perfluorooctane sulfonamide	DiMeFOSA	213181-78-3	0.93
2,2,3,3,4,4-hexafluoropentane-1,5-diol		376-90-9	-6.74
Perfluoro-1-octanol		114292-89-6	-0.44
5H-Octafluoropentanoyl fluoride		813-03-6	1.73
Octafluorobutan-1-one		335-42-2	2.71
Perfluorobutanal		375-02-0	1.17
Perfluoroglutaryl fluoride		678-78-4	2.23
[4-(Heptadecafluorooctyl)phenyl]methanol		163114-33-8	-0.92
4-(Heptadecafluorooctyl)phenol		80804-82-6	-1.26
(Perfluoro-5-methylhexyl)ethyl 2-methylprop-2-enoate		50836-66-3	1.29
1H-Perfluoro-1,1-propanediol		422-63-9	-5.11
1H,1H-Perfluorooctyl acrylate		307-98-2	2.11
Hexafluoroglutaryl chloride		678-77-3	1.47
(Heptafluoropropyl)trimethylsilane		3834-42-2	2.31

Bis(1H,1H-perfluoropropyl)amine		883498-76-8	0.91
Perfluoro-1-octanesulfonyl chloride		423-60-9	3.96
Methyl perfluoro(3-(1-ethenyloxypropan-2-yloxy)propanoate)		63863-43-4	3.04
2,2,3,3,4,4,4-Heptafluorobutyl methacrylate		13695-31-3	0.63
(Heptafluorobutanoyl)pivaloylmethane		17587-22-3	-1.01
Perfluorocyclohexanecarbonyl fluoride		6588-63-2	2.85
2-(Perfluorooctyl)ethanthiol		34143-74-3	2.66
1H,1H-Perfluoro-3,6,9-trioxadecan-1-ol		147492-57-7	1.10
1H,1H,7H-Perfluoroheptyl 4-methylbenzenesulfonate		424-16-8	-2.54
3-(Perfluorooctyl)propanol		1651-41-8	-0.43
1H,1H,8H,8H-Perfluoro-3,6-dioxaoctane-1,8-diol		129301-42-4	-5.57
1H,1H,7H-Dodecafluoro-1-heptanol		335-99-9	-2.11
1H,1H,5H-Perfluoropentanol		355-80-6	-2.74
Methyl 2H,2H,3H,3H-perfluoroheptanoate		132424-36-3	-0.10
1H,1H,11H,11H-Perfluorotetraethylene glycol		330562-44-2	-4.17
1H,1H,8H,8H-Perfluorooctane-1,8-diol		90177-96-1	-6.05
2-Aminohexafluoropropan-2-ol		31253-34-6	-3.41
1-Iodo-1H,1H,2H,2H-perfluoroheptane		1682-31-1	1.29
1-Bromopentadecafluoroheptane		375-88-2	4.06
1-(Perfluorohexyl)octane		133331-77-8	4.06
Dimethoxymethyl((perfluorohexyl)ethyl)silane		85857-17-6	1.68
Triethoxy((perfluorohexyl)ethyl)silane		51851-37-7	1.72
2,2,2-Trifluoroethyl perfluorobutanesulfonate		79963-95-4	1.46
(Perfluorobutyryl)-2-thenoylmethane		559-94-4	-2.70
tris(Trifluoroethoxy)methane		58244-27-2	0.57
Trichloro((perfluorohexyl)ethyl)silane		78560-45-9	2.93
Heptafluorobutyl iodide		374-98-1	0.49
2-(Trifluoromethoxy)ethyl trifluoromethanesulfonate		329710-76-1	-1.20
1H,1H,5H,5H-Perfluoro-1,5-pentanediol diacrylate		678-95-5	-2.58
Dichloromethyl((perfluorohexyl)ethyl)silane		73609-36-6	2.78
3H-Perfluoro-2,2,4,4-tetrahydroxypentane		77953-71-0	-8.52
1H,1H,9H-Perfluorononyl acrylate		4180-26-1	1.05
2,2-Difluoroethyl triflate		74427-22-8	-1.28
3H,3H-Perfluoro-2,4-hexanedione		20825-07-4	0.67
1H,1H,10H,10H-Perfluorodecane-1,10-diol		754-96-1	-5.33
2,2,3,3-Tetrafluoropropyl acrylate		7383-71-3	-1.20
1H,1H,6H,6H-Perfluorohexane-1,6-diol diacrylate		2264-01-9	-1.94
Perfluorooct-1-ene		559-14-8	4.54
6:1 Fluorotelomer alcohol		375-82-6	-0.67
3-(Perfluoro-3-methylbutyl)-1,2-propenoxide		54009-81-3	0.38
1H,1H-Heptafluorobutyl epoxide		1765-92-0	-0.17
Ethyl pentafluoropropionyl acetate		663-35-4	-1.03
3,3-Bis(trifluoromethyl)-2-propenoic acid		1763-28-6	-3.67
3-(Perfluorohexyl)-1,2-epoxypropane		38565-52-5	1.09
N-Methyl-N-trimethylsilylheptafluorobutyramide		53296-64-3	0.37
Trifluoroacetic acid	TFA	76-05-1	-3.77
Perfluoropropionic acid	PFPrA	422-64-0	-3.35
Perfluorobutanoic acid	PFBA	375-22-4	-2.93

Perfluoropentanoic acid	PFPeA	2706-90-3	-2.55
Perfluorohexanoic acid	PFHxA	307-24-4	-2.12
Perfluoroheptanoic acid	PFHpA	375-85-9	-1.79
Perfluorooctanoic acid	PFOA	335-67-1	-1.41
Perfluorononanoic acid	PFNA	375-95-1	-0.97
Perfluorodecanoic acid	PFDA	335-76-2	-0.53
Perfluoroundecanoic acid	PFUnDA	2058-94-8	-0.15
Perfluorododecanoic acid	PFDoDA	307-55-1	0.31
Perfluorotridecanoic acid	PFTrDA	72629-94-8	0.64
Perfluorotetradecanoic acid	PFTeDA	376-06-7	1.03
Perfluoropentadecanoic acid	PFPeDA	141074-63-7	1.42
Perfluorohexadecanoic acid	PFHxDA	67905-19-5	1.92
Perfluoroheptadecanoic acid	PFHpDA	57475-95-3	2.32
Perfluorooctadecanoic acid	PFODA	16517-11-6	2.69
Perfluorononadecanoic acid	PFNDA	133921-38-7	3.28
Perfluoroeicosanoic acid	PFEiA	68310-12-3	3.73
2-(Perfluorobutyl)ethanoic acid	4:2 FTCA		-3.25
2-(Perfluorohexyl)ethanoic acid	6:2 FTCA	53826-12-3	-2.39
2-(Perfluorooctyl)ethanoic acid	8:2 FTCA	27854-31-5	-1.63
2-(Perfluorodecyl)ethanoic acid	10:2 FTCA	53826-13-4	-0.81
2H,2H,3H,3H-Perfluorohexanoic acid	3:3 FTCA	356-02-5	-3.50
2H,2H,3H,3H-Perfluorooctanoic acid	5:3 FTCA	914637-49-3	-2.75
2H,2H,3H,3H-Perfluorodecanoic acid	7:3 FTCA	812-70-4	-1.81
(Z)-2H-Perfluoro-2-hexenoic acid	Z-4:2 FTUCA		-2.68
(E)-2H-Perfluoro-2-hexenoic acid	E-4:2 FTUCA		-2.69
(Z)-2H-Perfluoro-2-octenoic acid	Z-6:2 FTUCA	70887-88-6 (racemic)	-1.86
(E)-2H-Perfluoro-2-octenoic acid	E-6:2 FTUCA	70887-88-6 (racemic)	-1.97
(Z)-2H-Perfluoro-2-decenoic acid	Z-8:2 FTUCA	70887-84-2 (racemic)	-0.99
(E)-2H-Perfluoro-2-decenoic acid	E-8:2 FTUCA	70887-84-2 (racemic)	-1.05
(Z)-2H-Perfluoro-2-dodecenoic acid	Z-10:2 FTUCA	70887-94-4 (racemic)	-0.26
(E)-2H-Perfluoro-2-dodecenoic acid	E-10:2 FTUCA	70887-94-4 (racemic)	-0.34
Perfluorooctane sulfonamidoacetic acid	FOSAA	2806-24-8	-5.41
N-Methylperfluorooctane sulfonamidoacetic acid	MeFOSAA	2355-31-9	-3.85
N-Ethylperfluorooctane sulfonamidoacetic acid	EtFOSAA	2991-50-6	-3.98
2,3,3,3-Tetrafluoro-2-(1,1,2,2,3,3,3-heptafluoropropoxy)propanoic acid	HFPO-DA	13252-13-6	-1.43
4,8-Dioxa-3H-perfluorononanoic acid	ADONA	919005-14-4	-1.45
Perfluoro(2-ethoxyethane)sulfonic acid	PFEESA	113507-82-7	-3.49
Perfluoro-3,6-dioxaheptanoic acid	NFDHA	151772-58-6	-1.81
Perfluoro-3-methoxypropanoic acid	PFMPA	377-73-1	-2.67
Perfluoro(4-methoxybutanoic) acid	PFMBA	863090-89-5	-2.28
Trifluoromethanesulfonic acid	TFSA	1493-13-6	-5.53
Pentafluoroethanesulfonic acid	PFES	354-88-1	-4.98
Perfluoropropanesulfonic acid	PFPrS	423-41-6	-4.65

Perfluorobutanesulfonic acid	PFBS	375-73-5	-4.21
Perfluoropentanesulfonic acid	PFPeS	2706-91-4	-3.82
Perfluorohexanesulfonic acid	PFHxS	355-46-4	-3.36
Perfluoroheptanesulfonic acid	PFHpS	375-92-8	-3.02
Perfluorooctanesulfonic acid	PFOS	1763-23-1	-2.73
Perfluorononanesulfonic acid	PFNS	68259-12-1	-2.18
Perfluorodecanesulfonic acid	PFDS	335-77-3	-1.83
Perfluoro-4-ethylcyclohexanesulfonic acid	PFECHS	646-83-3	-4.10
2-(Perfluorobutyl)ethane-1-sulfonic acid	4:2 FTS	757124-72-4	-6.57
2-(Perfluorohexyl)ethane-1-sulfonic acid	6:2 FTS	27619-97-2	-5.66
2-(Perfluorooctyl)ethane-1-sulfonic acid	8:2 FTS	39108-34-4	-4.85
2-(Perfluorodecyl)ethane-1-sulfonic acid	10:2 FTS	120226-60-0	-4.05
9-Chlorohexadecafluoro-3-oxanonane-1-sulfonic acid	9Cl-PF3ONS	756426-58-1	-2.45
11-Chloroeicoafluoro-3-oxaundecane-1-sulfonic acid	11Cl-PF3OUdS	83329-89-9	-1.58
Mono[2-(perfluorohexyl)ethyl] hydrogen phosphate	6:2 monoPAP	57678-01-0	-9.96
Mono[2-(perfluorooctyl)ethyl] hydrogen phosphate	8:2 monoPAP	57678-03-2	-9.62
Bis[2-(perfluorohexyl)ethyl] hydrogen phosphate	6:2 diPAP	57677-95-9	-3.36
(Perfluorohexyl)ethyl (perfluorooctyl)ethyl hydrogen phosphate	6:2/8:2 diPAP	943913-15-3	-2.66
Bis[2-(perfluorooctyl)ethyl] hydrogen phosphate	8:2 diPAP	678-41-1	-1.40
Bis(perfluorohexyl)phosphinic acid	6:6 PFPi	70609-44-8	-0.70
(perfluorooctyl)(perfluorohexyl)phosphinic acid	6:8 PFPi	610800-34-5	0.07
Perfluoro-1-methylheptane sulfonic acid	P1MHpS		-2.92
Perfluoro-3,5,5-trimethylhexanoic acid	P355TMHxA		-2.07
Perfluoro-3,5-dimethylhexanoic acid	P35DMHxA		-1.98
Perfluoro-3,5-dimethylhexane sulfonic acid	P35DMHxS		-3.38
Perfluoro-3,7-dimethyloctanoic acid	P37DMOA		-1.15
Perfluoro-3-methylheptanoic acid	P3MHpA		-1.62
Perfluoro-3-methylheptane sulfonic acid	P3MHpS		-3.04
Perfluoro-4,4-dimethylhexanoic acid	P44DMHxA		-2.34
Perfluoro-4,4-dimethylhexane sulfonic acid	P44DMHxS		-3.54
Perfluoro-4,5-dimethylhexanoic acid	P45DMHxA		-2.18
Perfluoro-4,5-dimethylhexane sulfonic acid	P45DMHxS		-3.41
Perfluoro-4-methylheptanoic acid	P4MHpA		-1.89
Perfluoro-4-methylheptane sulfonic acid	P4MHpS		-2.98
Perfluoro-5,5-dimethylhexanoic acid	P55DMHxA		-2.23
Perfluoro-5,5-dimethylhexane sulfonic acid	P55DMHxS		-3.25
Perfluoro-5-methylheptanoic acid	P5MHpA		-1.84
Perfluoro-5-methylheptane sulfonic acid	P5MHpS		-3.02
Perfluoro-6-methylheptanoic acid	P6MHpA		-1.74
Perfluoro-6-methylheptane sulfonic acid	P6MHpS		-3.00
Perfluoro-7-methyloctanoic acid	ipPFNA		-1.33
Perfluoro-7-methyloctane sulfonic acid	ipPFNS		-2.55

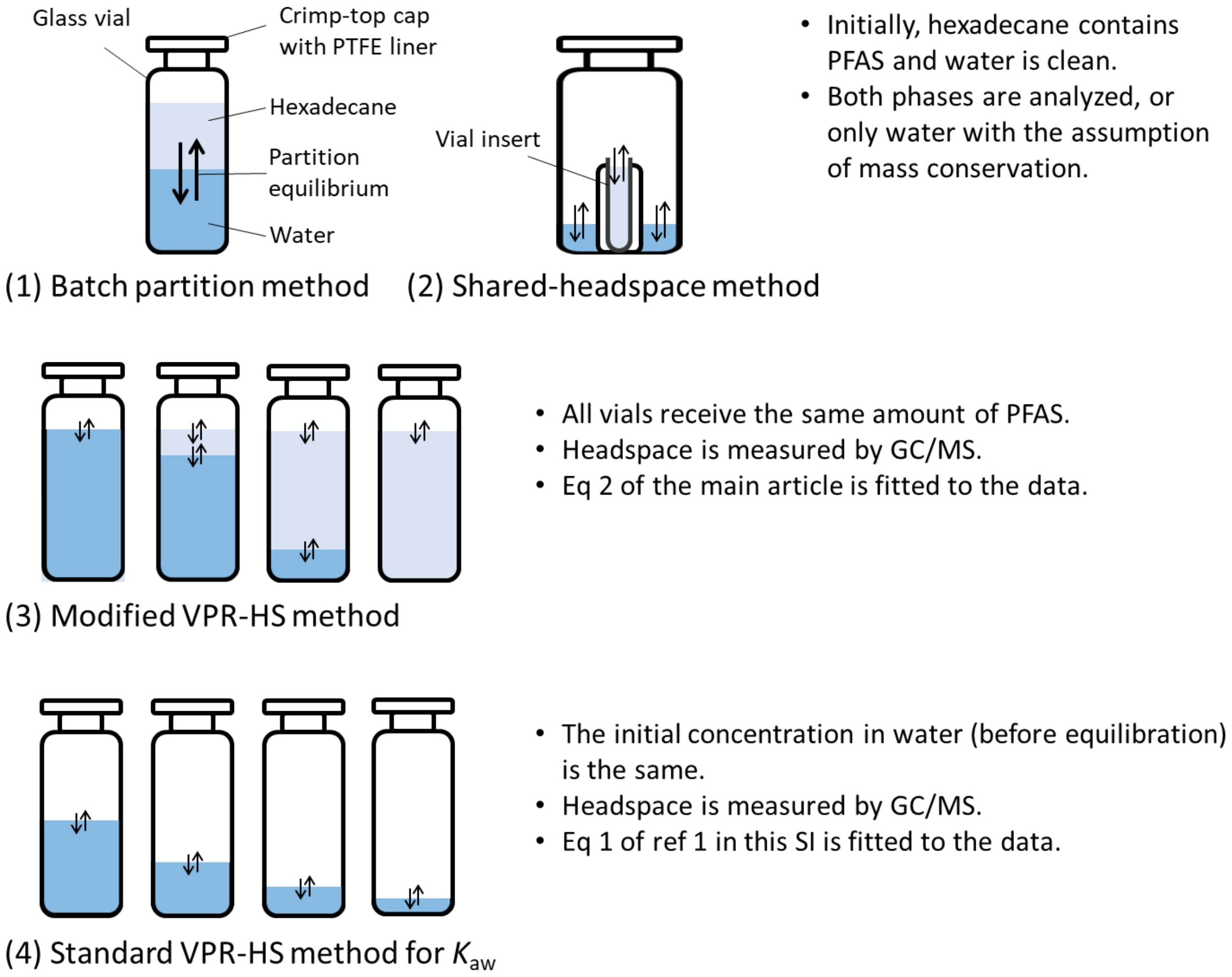

Figure S1. Schematic illustration of experimental methods for the determination of partition coefficients. (1), (2), and (3) are for $K_{Hxd/w}$, and (4) is for K_{aw}.

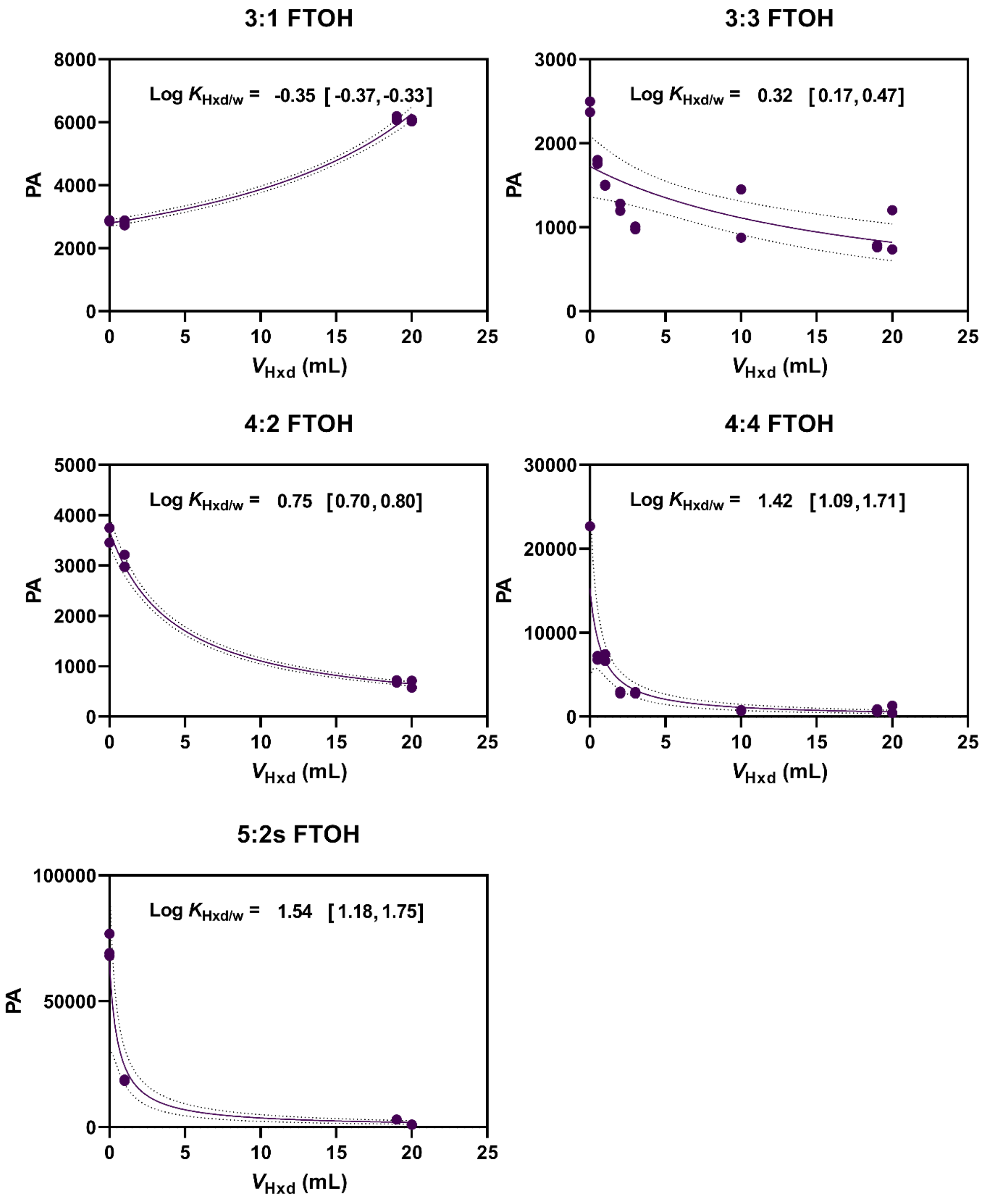

Figure S2. The results of log $K_{Hxd/w}$ determination by the modified VPR-HS method. The mean and its 95% CIs of log $K_{Hxd/w}$ obtained by model fitting are shown within the figure. The solid line indicates the model fit, and the dotted lines the 95% CIs of model fit.

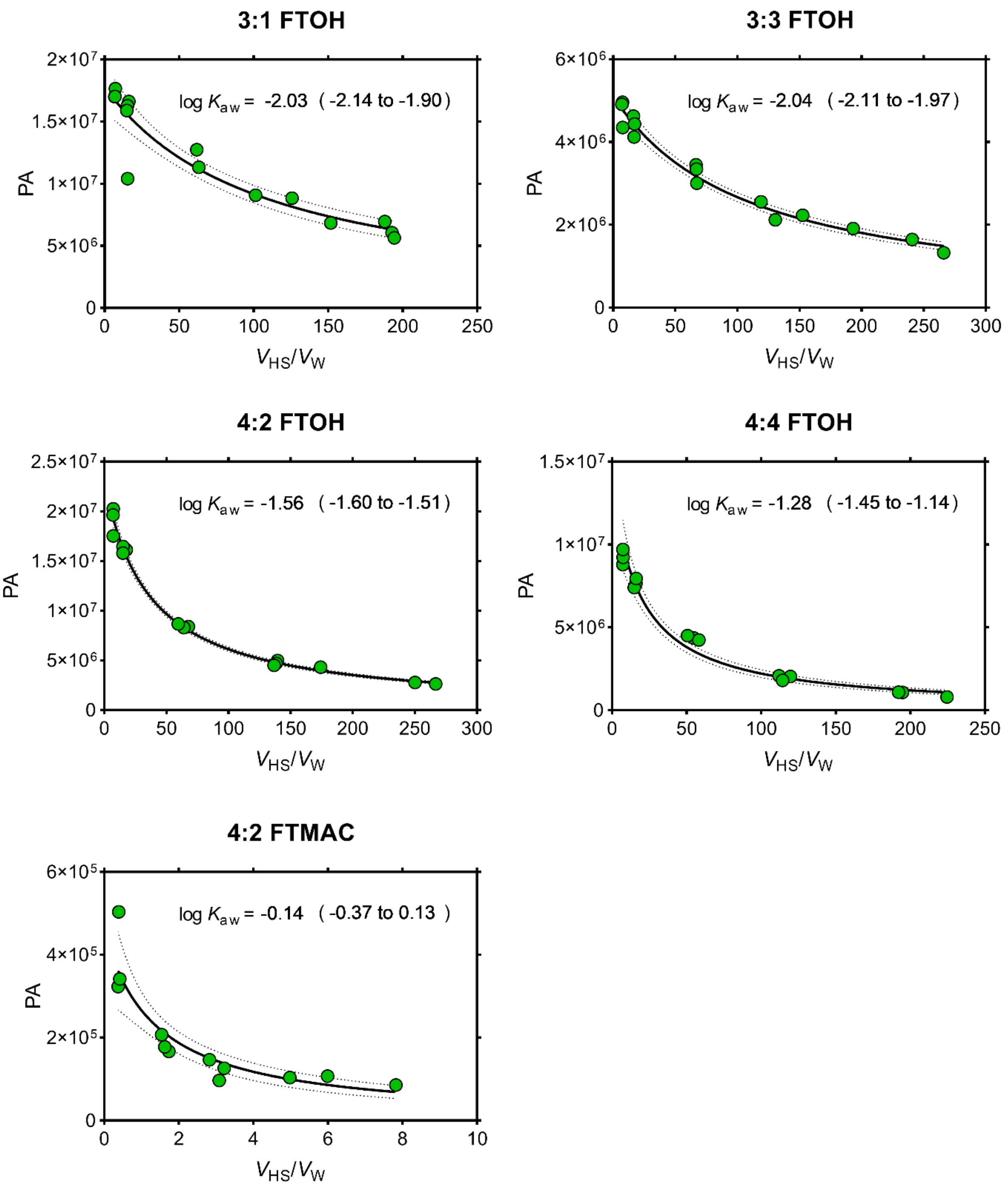

Figure S3. The results of log K_{aw} determination by the standard VPR-HS method. The mean and its 95% CIs of log K_{aw} obtained by model fitting are shown within the figure. The solid line indicates the model fit, and the dotted lines the 95% CIs of model fit.

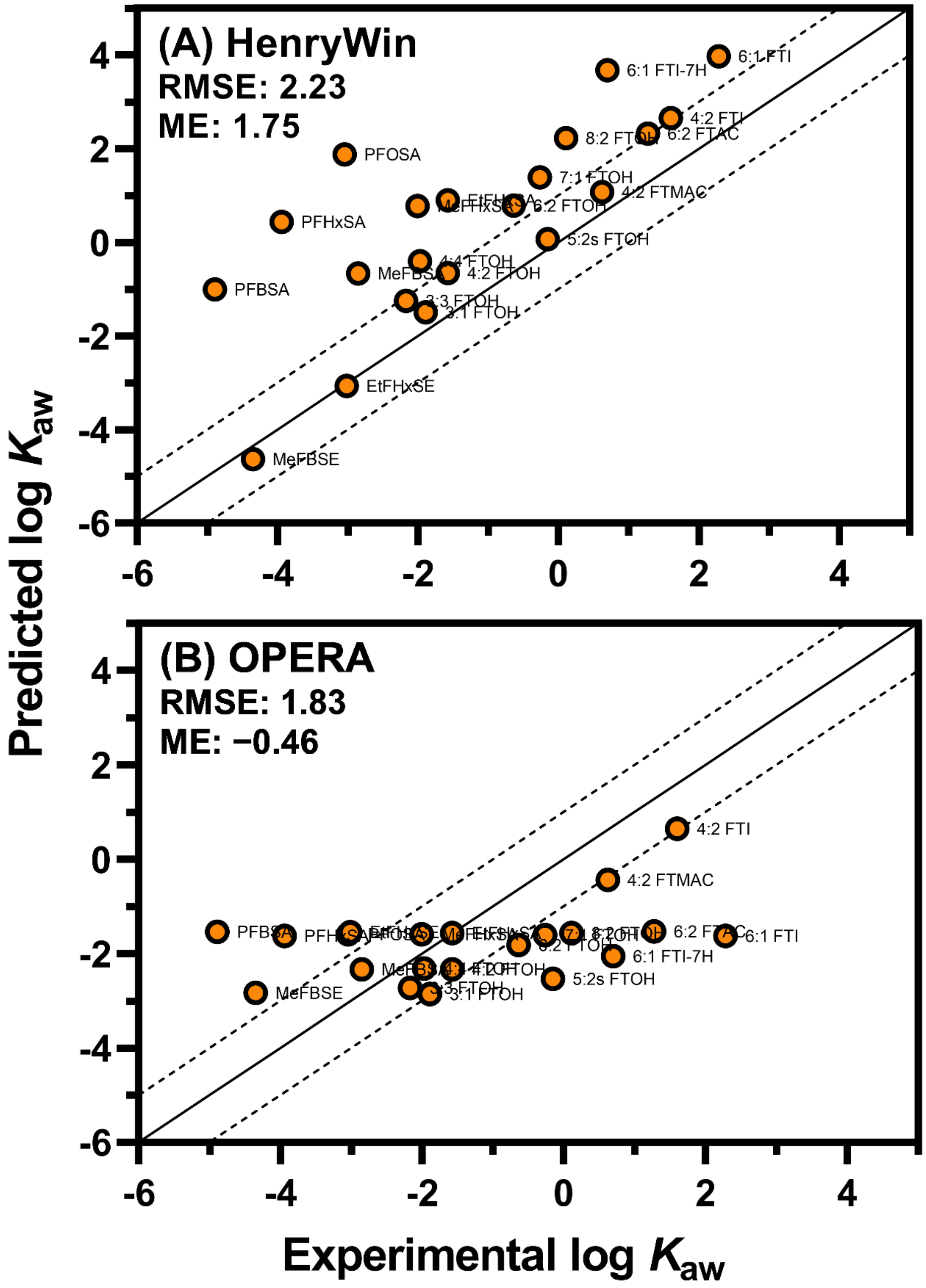

Figure S4. Predicted vs experimental log K_{aw} (enlarged version of Figure 2 in the main manuscript).

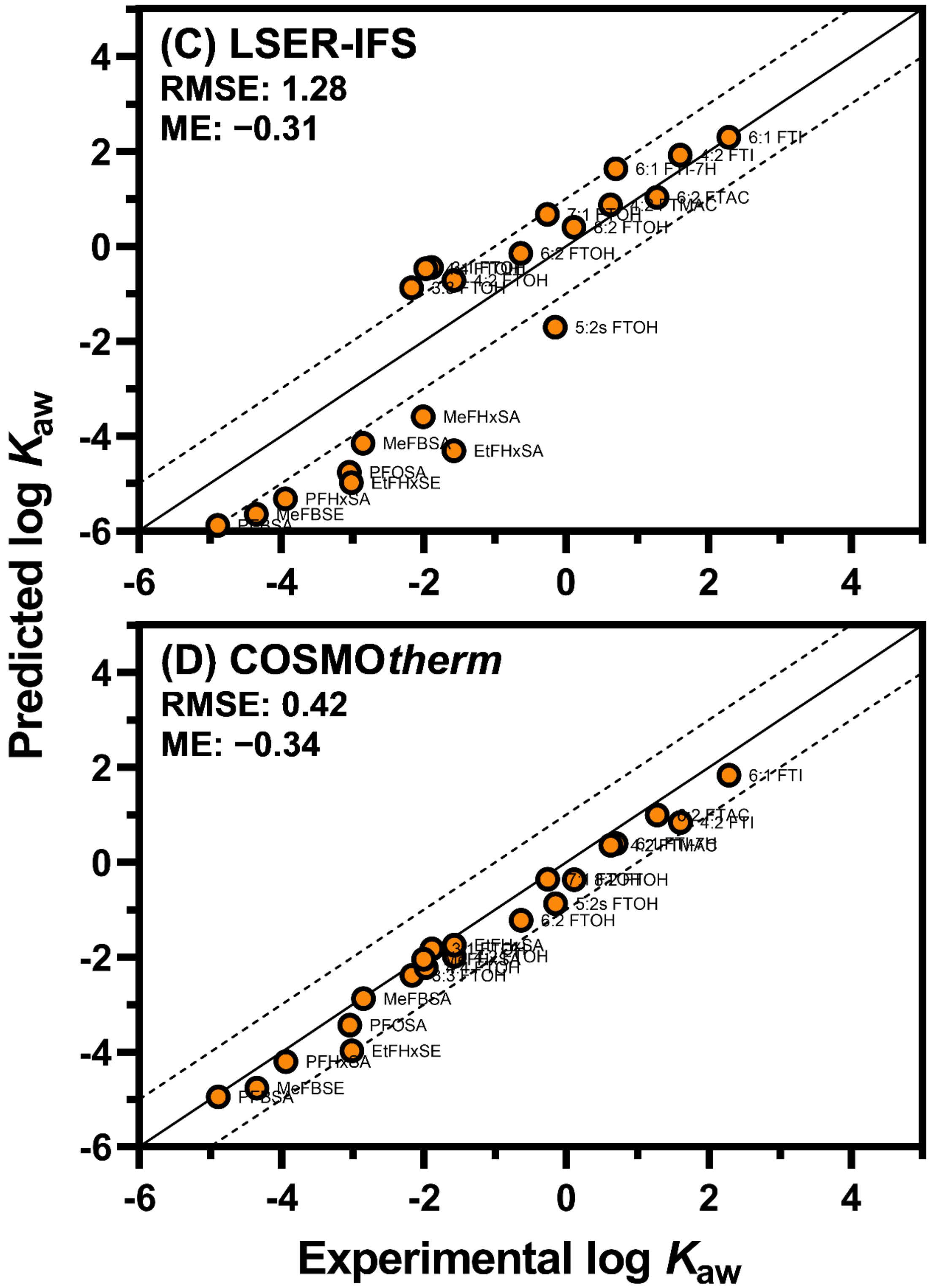

Figure S4. (continued)

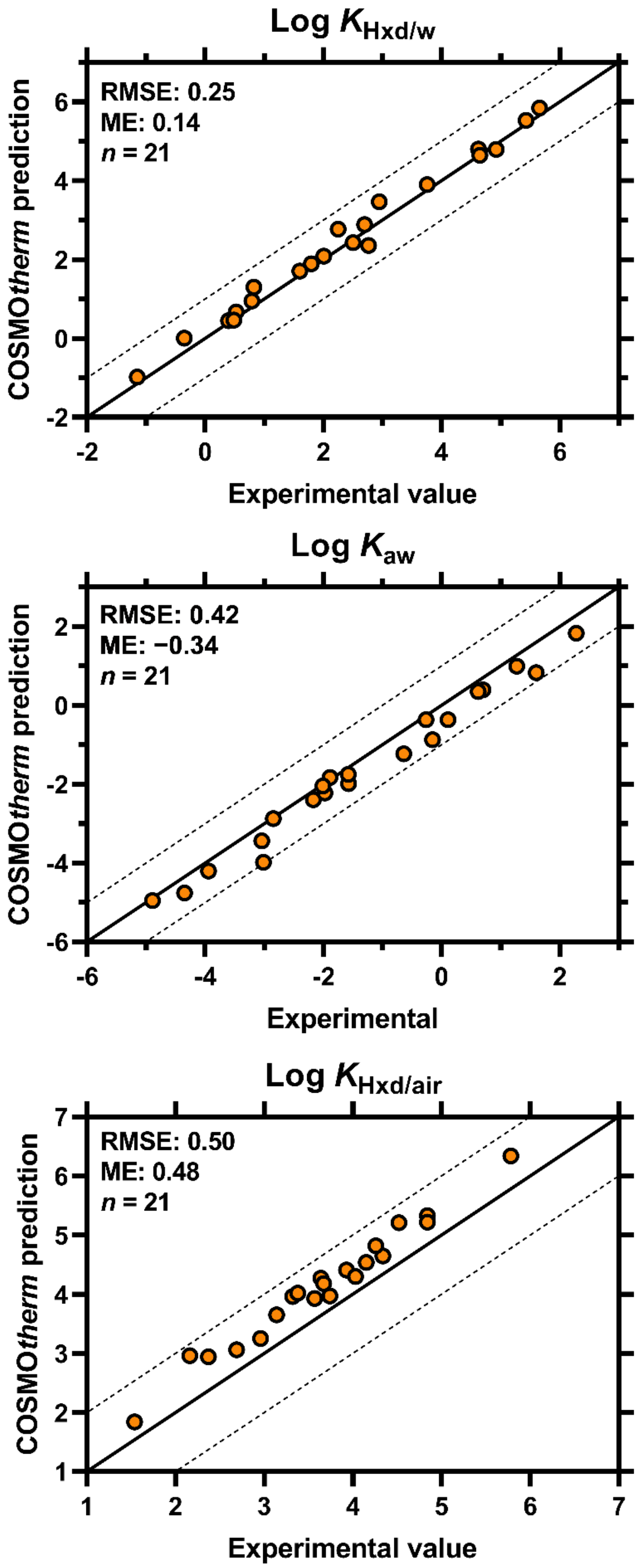

Figure S5. COSMO*therm*-predicted vs experimental values for log $K_{Hxd/w}$, log K_{aw}, and log $K_{Hxd/air}$. The solid lines indicate the 1:1 agreement, and dashed lines 1 log unit deviations.

References

1. Hammer, J.; Endo, S. Volatility and nonspecific van der Waals interaction properties of per- and polyfluoroalkyl substances (PFAS): Evaluation using hexadecane/air partition coefficients. *Environ. Sci. Technol.* **2022,** *56* (22), 15737-15745.
2. Robbins, G. A.; Wang, S.; Stuart, J. D. Using the static headspace method to determine Henry's law constants. *Anal. Chem.* **1993,** *65* (21), 3113-3118.
3. Ettre, L. S.; Welter, C.; Kolb, B. Determination of gas-liquid partition coefficients by automatic equilibrium headspace-gas chromatography utilizing the phase ratio variation method. *Chromatographia* **1993,** *35* (1-2), 73-84.
4. Lei, Y. D.; Baskaran, S.; Wania, F. Measuring the octan-1-ol air partition coefficient of volatile organic chemicals with the variable phase ratio headspace technique. *J. Chem. Eng. Data* **2019,** *64* (11), 4793-4800.
5. Abusallout, I.; Holton, C.; Wang, J.; Hanigan, D. Henry’s law constants of 15 per- and polyfluoroalkyl substances determined by static headspace analysis. *J. Hazard. Mater. Lett.* **2022,** *3*, 100070.
6. Goss, K.-U.; Bronner, G.; Harner, T.; Hertel, M.; Schmidt, T. C. The partition behavior of fluorotelomer alcohols and olefins. *Environ. Sci. Technol.* **2006,** *40*, 3572-3577.
